# Statin Effects on Aggression: Results from the UCSD Statin Study, a Randomized Control Trial

**DOI:** 10.1371/journal.pone.0124451

**Published:** 2015-07-01

**Authors:** Beatrice A. Golomb, Joel E. Dimsdale, Hayley J. Koslik, Marcella A. Evans, Xun Lu, Steven Rossi, Paul J. Mills, Halbert L. White, Michael H. Criqui

**Affiliations:** 1 Department of Medicine, University of California San Diego, San Diego, CA, United States of America; 2 Department of Family and Preventive Medicine, University of California San Diego, San Diego, CA, United States of America; 3 Department of Psychiatry, University of California San Diego, San Diego, CA, United States of America; 4 Department of Economics, University of California San Diego, San Diego, CA, United States of America; 5 Department of Anesthesiology, University of California San Diego, San Diego, CA, United States of America; Clermont Université, FRANCE

## Abstract

**Background:**

Low/ered cholesterol is linked to aggression in some study designs. Cases/series have reported reproducible aggression increases on statins, but statins also bear mechanisms that could reduce aggression. Usual statin effects on aggression have not been characterized.

**Methods:**

1016 adults (692 men, 324 postmenopausal women) underwent double-blind sex-stratified randomization to placebo, simvastatin 20mg, or pravastatin 40mg (6 months). The Overt-Aggression-Scale-Modified–Aggression-Subscale (OASMa) assessed behavioral aggression. A significant sex-statin interaction was deemed to dictate sex-stratified analysis. Exploratory analyses assessed the influence of baseline-aggression, testosterone-change (men), sleep and age.

**Results:**

The sex-statin interaction was significant (P=0.008). In men, statins tended to decrease aggression, significantly so on pravastatin: difference=-1.0(SE=0.49)P=0.038. Three marked outliers (OASMa-change ≥40 points) offset otherwise strong significance-vs-placebo: statins:-1.3(SE=0.38)P=0.0007; simvastatin:-1.4(SE=0.43)P=0.0011; pravastatin:-1.2(SE=0.45)P=0.0083. Age≤40 predicted greater aggression-decline on statins: difference=-1.4(SE=0.64)P=0.026. Aggression-*protection* was emphasized in those with low baseline aggression: age<40-and-low-baseline-aggression (N=40) statin-difference-vs-placebo=-2.4(SE=0.71)P=0.0016. Statins (especially simvastatin) lowered testosterone, and increased sleep problems. Testosterone-drop on statins predicted aggression-decline: β=0.64(SE=0.30)P=0.034, particularly on simvastatin: β=1.29(SE=0.49)P=0.009. Sleep-worsening on statins significantly predicted aggression-increase: β=2.2(SE=0.55)P<0.001, particularly on simvastatin (potentially explaining two of the outliers): β=3.3(SE=0.83)P<0.001. Among (postmenopausal) women, a borderline aggression-increase on statins became significant with exclusion of one younger, surgically-menopausal woman (N=310) β=0.70(SE=0.34)P=0.039. The increase was significant, without exclusions, for women of more typical postmenopausal age (≥45): (N=304) β=0.68(SE=0.34)P=0.048 – retaining significance with modified age-cutoffs (≥50 or ≥55). Significance was observed separately for simvastatin. The aggression-*increase* in women on statins was stronger in those with low baseline aggression (N=175) β=0.84(SE=0.30)P=0.006. No statin effect on whole blood serotonin was observed; and serotonin-change did not predict aggression-change.

**Conclusion:**

Statin effects on aggression differed by sex and age: Statins generally decreased aggression in men; and generally increased aggression in women. Both findings were selectively prominent in participants with low baseline aggression – bearing lower change-variance, rendering an effect more readily evident.

**Trial Registration:**

Clinicaltrials.gov NCT00330980

## Introduction

Low cholesterol has been linked to aggression and to violent death or “non-illness mortality” (i.e. death from suicide, homicide and accident) in many observational studies [1–9]. Lowered cholesterol has been linked to aggression in primate experimental studies and meta-analysis of pre-statin randomized trials that show increased violent death with non-statin lipid reduction [10–12]. Cholesterol supports cell energy and many forms of cell energy deficit are linked to aggression. However, statin use, via endothelial and other benefits, may improve cell energy—so effects on statins need not parallel effects of low cholesterol. Indeed, statins were not associated with increased violent death in a meta-analysis, and any trend was toward reduction [13]. Nonetheless, individual cases of reproducible aggression/irritability-increase have been reported with statins [14, 15]; and aggressive responding was found to be higher in women on lipid-lowering medications (in the statin era), adjusted for potential confounders [16]. Men and women differ in their risk of violence [2]; and in effects of statins on aggression-related physiological factors like testosterone [17]. Age also affects statin risk-benefit, and also aggression risk; non-illness mortality is the leading cause of death in those under age 40 [18].

The University of California, San Diego (**UCSD**) Statin Study sought to examine noncardiac effects of statins; aggression was a primary endpoint.

We sought to examine the relation of statins vs placebo to aggression, assessing for a sex-statin interaction. We sought also to assess potential mediators, examining whether change in testosterone or sleep—each reported to be influenced by statins [17, 19, 20], and each with a literature relation to aggression [21, 22]—related to change in aggression, on statins.

Randomization was stratified on sex, inclusion criteria differed by sex, and sex steroid products of cholesterol differ by sex, providing *a priori* grounds for possible gender differences in effects on aggression. For these reasons testosterone was assessed; and it was prespecified that sex-statin interaction effects, if significant, would dictate analysis stratified by sex [23]. Only post-menopausal women were included, leading to different ages of male and female participants, which adds importance to examination of age effects, particularly if sex-differential effects are identified.

Animal studies and other evidence have suggested a relationship between low/lower cholesterol, reduced central serotonin and aggression [1, 24, 25]. Whole blood serotonin, which can be assessed without invasive procedures, can have an inverse relationship to central serotonin, and whole blood serotonin has been reported to be higher in aggressive young men (but not young women) [26]; however in a small sample of children, an opposite relationship to aggression was reported [27]. Therefore we sought to examine whether statins reduced whole blood serotonin, and/or whether changes in whole blood serotonin related to changes in aggression.

Testosterone, a steroid hormone, is a product of the mevalonate pathway inhibited by statins (S1 Fig). While variance is substantial, meta-analysis as well as larger individual studies (including this one) have reported that simvastatin modestly but significantly reduces testosterone on average [17, 19, 28]. Statin use might also be viewed as affording the opportunity to examine whether experimental inhibition of the rate-limiting step in the mevalonate pathway [29, 30] influences aggression; and/or whether there is a relationship between the magnitude of testosterone-change and aggression-change unexplained by changes in low density lipoprotein cholesterol (**LDL**).

We previously reported that simvastatin but not pravastatin significantly increased sleep problems relative to placebo [20]. Sleep problems and sleep apnea (which has been reported as an apparent effect of simvastatin [31]) have been linked to aggression and irritability [21, 32–34]. Examining whether sleep effects contribute, as mediators, to simvastatin effects on aggression (if any) is also of interest.

## Methods

### Design and intervention

The UCSD Statin Study was a parallel-design randomized double-blind placebo-controlled clinical trial with equal (1/3) probability of randomization to simvastatin 20mg, pravastatin 40mg or placebo (microcrystalline cellulose) in identical blinding capsules for six months. Randomization was stratified by sex. The study was active from 2000–2005. The trial ended only after all participants were recruited, scheduled and seen, and their participation was complete.

### Setting and participants

Details on the time course of study recruitment are provided elsewhere [35]. Sample size was chosen to provide ability to detect ~0.25 standard deviation (**SD**) effect on aggression among men in the sample with a power of 90% (this recognized that effects may differ in men and women, but powered only for the larger i.e. male group). Participants were 1016 community-dwelling adults from Southern California, comprising 692 men age ≥20 and 324 surgically or chronologically postmenopausal women. Eligibility required absence of known cardiovascular disease, current cancer (except nonmelanoma skin cancer), HIV, or diabetes mellitus. Measured screening fasting blood glucose >142mg/dL, or LDL <115mg/dL (2.98mmol/L) or >190mg/dL (4.92mmol/L) also led to exclusion. The admissible LDL range was selected to exclude individuals with very high LDL or low LDL (based on standards at the time of the study proposal), in whom randomization to placebo or statin, respectively, might be thought unethical.

### Ethics statement

The study protocol was approved by the UCSD Human Research Protections Program (**HRPP**), and all participants gave written informed consent to participate. Documentation for the original study protocol is unavailable. (The study commenced in 1999, many materials were disposed of during a subsequent move, and the HRPP did not retain a copy.) All participants were seen at UCSD. The data & safety monitoring board (**DSMB**) provided independent oversight of the study.

### Randomization

A computer-generated, blocked randomization sequence with blocksize 20, stratified on sex, was designed by the study statistician (Halbert L. White, PhD) and provided to the study pharmacist (Stephen Funk, PharmD) who used the sequence to match assigned treatment to sequentially numbered bottles. Sequential participants who met eligibility criteria and gave informed consent were enrolled by the study coordinator (or study staff under her supervision), assigned sequential study identification numbers, and received the bottle with the corresponding number. The randomization sequence, and translation between sequential study ID and randomization assignment, was preserved offsite on non-networked computers, with access only by the study pharmacist and the data manager, both with offsite offices. (The data manager did not interact with subjects during their participation nor assign outcomes, but had confidential interactions with the DSMB; access allowed for analyses as requested by the DSMB.) With the exception of confidential requests, if any, by the DSMB unknown to investigators and other study personnel, no unblinding of treatment assignment occurred until all participants had completed the randomized treatment period. Thus, no participants, and no study staff who interacted with participants, had any access to randomization information throughout the period of the study.

Study staff received sequentially numbered treatment bottles from the pharmacy (with no knowledge of randomization sequence). Sequentially numbered “male” and “female” bottles were given to sequentially eligible male and female participants. All participants, study staff, and investigators were blinded to randomization assignment throughout subjects’ participation.

### Outcomes, variables and follow-up

Aggression was a primary outcome for the study. The Point Subtraction Aggression paradigm (**PSAP**) first-session, which had shown favorable psychometric properties and good relation to predictors of aggression and to other measures of aggression (convergent validity) in cross-sectional analysis in young participants with a single tester [36], was the originally designated primary aggression outcome. However, cross-sectional analysis of baseline data from screenees and participants, intended to confirm that these favorable properties were maintained, instead showed that the PSAP, which relies on deception, retained none of these indicators of validity in this older study sample with multiple testers. For this reason, the DSMB approved modification of the primary behavioral aggression endpoint to the aggression subscale of the Overt-Aggression-Scale-Modified(**OASMa**) [37, 38], which retained the focus on behaviors, and was assessed in all participants at baseline (this determination was based on baseline data only, with no unblinding or examination of on-treatment values). OASMa effects were assessed by change from baseline to the final on-treatment visit. This validated instrument assesses actual behavioral aggression during the previous week, including verbal assaults, assaults against objects, assaults against others, and assaults against self. Any positive value signifies that actual behavioral aggression of some form occurred in the prior week, and thus has clinical relevance. Scoring is accomplished by summing events in each category, weighting events by their severity. Whole blood serotonin was also measured, and was a primary outcome. (Additional cognitive outcomes will be presented separately.)

### Potential mediating variables

Whole blood serotonin (nM) was assessed at baseline and on treatment, as above. The methods employed for the whole blood serotonin are described elsewhere [39]. Circulating levels of total testosterone, from blood drawn at baseline and the final on-treatment (six month), were assessed in duplicate by radioimmunoassay (Diagnostic Systems Laboratories, Houston, TX). Inter- and intra-coefficients of variation were <10%; assay sensitivity was 0.08 ng/mL. Lipids were also measured.

Sleep problems were self-rated at baseline (0–10) and as change-from-baseline on follow-up, on a 5-point Likert scale from much worse to much better (than at baseline). These self-ratings showed strong convergent validity (both for baseline and change rating) with numerous subjective and objective outcomes to which adverse sleep is known to be associated. (We have reported, for instance, that worsening of sleep problems on simvastatin significantly predicted rise in glucose [40] and increase in weight on simvastatin [41], consistent with the literature in which statins [42–44], and sleep problems [40, 45–49], promote dysglycemia and increased weight. (Mitochondrial effects of statins [50] provide one potential common cause [47, 51].)

### Statistical analyses

Power/effect size calculations were designed to identify a 0.25 SD effect in the total sample. Analyses were conducted using Stata version 8.0 and 11.0 (Stata Corporation, College Station, Texas).

Assessment for baseline comparability was undertaken, using t-tests of difference in mean.

For those with on-treatment values, use of last-(on-treatment)-value-carried-forward analysis was the prespecified approach. On-treatment visits occurred at one month (full visit) and three months (blood draw visit only) as well as six months.

Regression was conducted to assess for significance of a sex-statin interaction. Significance of a sex-statin interaction term (adjusted for both components of the interaction) was prespecified to lead to sex-stratified analysis.

T-tests were the primary analysis modality to compare treatment effects (OASMa change) on statin vs placebo. Regression analyses was employed in selected settings, such as to permit adjustment for baseline values where there were baseline disparities, to examine interaction terms, and to examine mediation (by serotonin, testosterone, or sleep; see below).

In men, presence of three influential outliers, inclusion of which materially altered findings, led to conduct of analyses with and without outliers. Both analyses are important. Assessment excluding outliers shows the “typical” or usual effect. Assessment including outliers shows the overall effect, and can underscore the magnitude of impact of the outliers, which themselves may be important.

Because these outliers so materially influenced the findings in men, two supplementary (exploratory) analyses were performed in men, to better appraise the nature of the statin effect on aggression in men. One examined the direction/sign of effect on aggression, which is insensitive to outliers, and one assessed the mean change from baseline, stratified by randomization group.

For both men and women, we evaluated effect modification based on baseline aggression. One reason is potential for effect modification *per se*. A second is that those with higher baseline aggression may have more fluctuations in aggression from sources independent of statins; the greater variance may relatively obscure an effect (even for comparable effect size) that may be evident in a lower change-variance subgroup.

Age has served as an effect modifier for numerous statin effects [44, 50, 52, 53], and exploratory analyses stratified by age were conducted in both men and women. Men under age 40 are the group at highest risk of violence [18], so to separately examine effects in this higher aggression risk group, stratification in men occurred at 40. Because only postmenopausal women were included, and female participants were older on average than men (by ~7 years), the same age cutpoint could not be used. Additionally, one purpose of stratification in women was somewhat different. Stratification at age 45 was performed, in order to exclude women with early and surgical menopause who may be physiologically different (and may respond differently to statins). Reassessments with cutoffs of 50 and 55 were conducted to assess for consistency of findings.

Regarding assessment of potential mediators, the correlation of baseline whole blood serotonin with baseline aggression was assessed, stratified by age and gender, to determine whether the literature-reported relationship was retained. We then assessed whether statins (vs placebo) affected serotonin, overall or stratified by sex and age. And we assessed whether change in serotonin on statins related to change in aggression, when/if any statin effect on serotonin was observed.

We previously reported that simvastatin significantly reduced testosterone in men (only); an effect that related significantly to drop in LDL [17]. Simvastatin is lipophilic, benefiting brain and testes penetration; moreover “simvastatin in addition to its known inhibitory effect on HMG-CoA reductase activity, also affects the later steps of testicular steroidogenesis by selectively inhibiting the 17-ketosteroid-oxidoreductase catalyzed conversion of dehydroepiandrosterone and androstenedione to androstenediol and testosterone respectively” [54].

Therefore we examined whether change in aggression on statins, or on placebo, related to change in testosterone (adjusted for baseline testosterone)—without and with adjustment for LDL (baseline and change). Since simvastatin (only) significantly increased sleep problems in this sample [20], regression analysis in men considering both testosterone and sleep problems was conducted. (This sought to assess whether testosterone drop may serve as a potential source of decline in aggression, particularly on simvastatin; and whether worsening sleep problems may concurrently serve as a potential source of increase in aggression, selectively on simvastatin.) Both change-value and baseline-value for each potential mediator were included in the regression models, stratified by treatment assignment.

## Results

Table 1 shows baseline characteristics of the sample, for the combined sample and stratified by sex. OASMa was comparable across randomization groups at baseline, though a trend to differences was observed in women.

**Table 1:** Baseline Comparability Across Randomization Arms (Mean ± SD*).

	Placebo	Statin Pooled	Pravastatin	Simvastatin	Statin comparisons to placebo
	*n = 342*	*n = 674*	*n = 338*	*n = 336*	
**Age (years)**	57.4 **±** 12.7	56.8 **±** 12.0	57.1 ± 12.0	56.6 ± 12.0	NS all
**Sex (% male)**	67.8	68.2	68.0	68.5	NS all
**Ethnicity (% white)**	81.9	80.9	81.7	80.1	NS all
**Smoker (% current)**	8.77	8.01	8.58	7.44	NS all
**Education (scaled 1–9)**	5.72 **±** 1.45	5.88 **±** 1.51	5.84 ± 1.47	5.91 ± 1.55	NS all
**TC (mg/dL)**	229 ± 27.8	229 **±** 30.2	232 ± 30.9	226 ± 29.2	NS all
**HDL (mg/dL)**	52.5 ± 15.4	52.0 ± 15.6	53.1 ± 16.2	50.8 ± 15.0	NS all
**Triglycerides (mg/dL)**	136 ± 79.6	136 ± 73.8	138 ± 75.1	135 ± 72.6	NS all
**LDL (mg/dL)**	150 ± 26.1	150 ± 25.1	152 ± 26.4	149 ± 23.6	NS all
**Ratio TC / HDL**	4.67 ± 1.24	4.73 ± 1.40	4.72 ± 1.56	4.75 ± 1.21	NS all
**Glucose (mg/dL)**	90.0 ± 9.08	90.2 ± 9.11	89.9 ± 8.65	90.5 ± 9.55	NS all
**SBP (mm Hg)**	126 ± 14.2	127 ± 14.4	127 ± 13.7	128 ± 15.0	NS all
**DBP (mm Hg)**	73.9 ± 8.79	75.2 ± 8.80	75.4 ± 8.98	75.0 ± 8.63	†
**Weight (lb)**	185 ± 41.0	185 ± 33.6	184 ± 33.7	186 ± 33.6	NS all
**Waist (cm)**	98.2 ± 13.8	98.0 ± 11.8	97.6 ± 11.6	98.3 ± 12.1	NS all
**Whole blood serotonin (nM)**	873 ± 335	868 ± 322	872 ± 342	864 ± 302	NS all
**Testosterone (ng/ml)**	3.07 ± 2.30	3.17 ± 2.31	3.18 ± 2.35	3.17 ± 2.28	NS all
**OASMa**	2.25 ± 5.26	2.76 ± 4.73	2.81 ± 5.19	2.71 ± 4.22	NS all

DBP = diastolic blood pressure; HDL = high density lipoprotein cholesterol; LDL = low density lipoprotein cholesterol; NS = nonsignificant; OASMa = Overt-Aggression-Scale-Modified–Aggression-Subscale; SBP = systolic blood pressure; TC = total cholesterol. Except items designated as %.

^†^ DBP: P = 0.04 placebo vs. statin; and P = 0.04 placebo vs. pravastatin.

NS (P>0.1) placebo vs. simvastatin; and simvastatin vs. pravastatin.

Weight and waist circumference were assessed at the screening visit (values shown are for randomized participants only). All other measures were assessed at the baseline visit. Conversion factors: To convert cholesterol (LDL, HDL, TC) from mg/mL to mmol/L, multiply by 0.0259. To convert triglyceride from mg/dL to mmol/L, multiply by 0.0113. To convert glucose from mg/dL to mmol/L, multiply by 0.0555. Baseline OASMa was not predicted by baseline testosterone: β = -0.020 (SE = 0.12) P = 0.87.

S2–S4 Figs (Consort) show participant retention by randomization arm, for all participants and for men and women separately. Reasons for participant drops are outlined in S1 Table. All participants with an on-treatment follow-up of any duration are included in the designated primary analysis, a last-(on-treatment)-value-carried-forward analysis.

LDL reductions were compatible with expectation (S2 Table). In this study sample, statins did not increase HDL-cholesterol, which dropped slightly in all arms (not significantly different on statins vs placebo). Of note, the mean drop in LDL was significantly greater on simvastatin than on pravastatin: P<0.0001 for the total sample, P = 0.0017 for men, P = 0.0021 for women.

Two deaths occurred during the study. One participant, on pravastatin, committed suicide. One participant, on placebo, was found deceased in his room and was designated as having had “heart failure” (no autopsy was undertaken). One relevant reportable adverse effect occurred. A woman dropped from the study at her husband’s urging at ~1 month into participation due to a reported marked adverse behavioral change. When unblinding was later undertaken, it was determined she had been on pravastatin. This participant communicated with us several years later, due to legal action emanating from the adverse behavioral change during her participation in the study—which reportedly led her to be fired from her job.

Table 2 shows results of a regression evaluating significance of a sex-statin interaction on aggression-change. A significant sex-interaction was affirmed, dictating (as prespecified) stratification of analysis by sex. Men whose aggression started out at zero (OASMa = 0) cannot show a further reduction in their aggression, but rather statins can attenuate upward fluctuations.

**Table 2:** Sex is a Significant Effect Modifier. Sex-by-Statin Interaction Term Significance.

	**All (Unrestricted by Baseline Aggression)**		
**Male x statin interaction term**			
	Statin vs placebo: N = 970	Simva vs placebo: N = 646	Prava vs placebo: N = 647
**Beta (SE)**	-1.2 (0.45)	-1.4 (0.54)	-0.93 (0.53)
**95%CI**	-2.1, -0.33	-2.5, -0.38	-2.0, 0.11
**P-value**	**0.007**	**0.008**	**0.079**
	**No Baseline Aggression (Baseline OASMa = 0)**		
**Male x statin interaction term**			
	Statin vs placebo: N = 514	Simva vs placebo: N = 344	Prava vs placebo: N = 364
**Beta (SE)**	-1.3 (0.38)	-1.1 (0.46)	-1.4 (0.50)
**95%CI**	-2.0, -0.53	-2.0, -0.21	-2.4, -0.45
**P-value**	**0.001**	**0.015**	**0.004**

Beta = regression coefficient; CI = confidence interval; OASMa = Overt-Aggression-Scale-Modified–Aggression-Subscale; Prava = pravastatin; SE = standard error; Simva = simvastatin. Regressions (robust SEs) adjust for baseline OASMa, as a primary source of change variance; the sex-interaction, and (as required) the individual components of the interaction term. Excludes outliers (OASMa absolute change >40 points). Without exclusion of outliers: Results lose significance for the full group. Results remain identical for the No Baseline Aggression group (all aggression outliers had nonzero baseline aggression).

**Fig 1:**
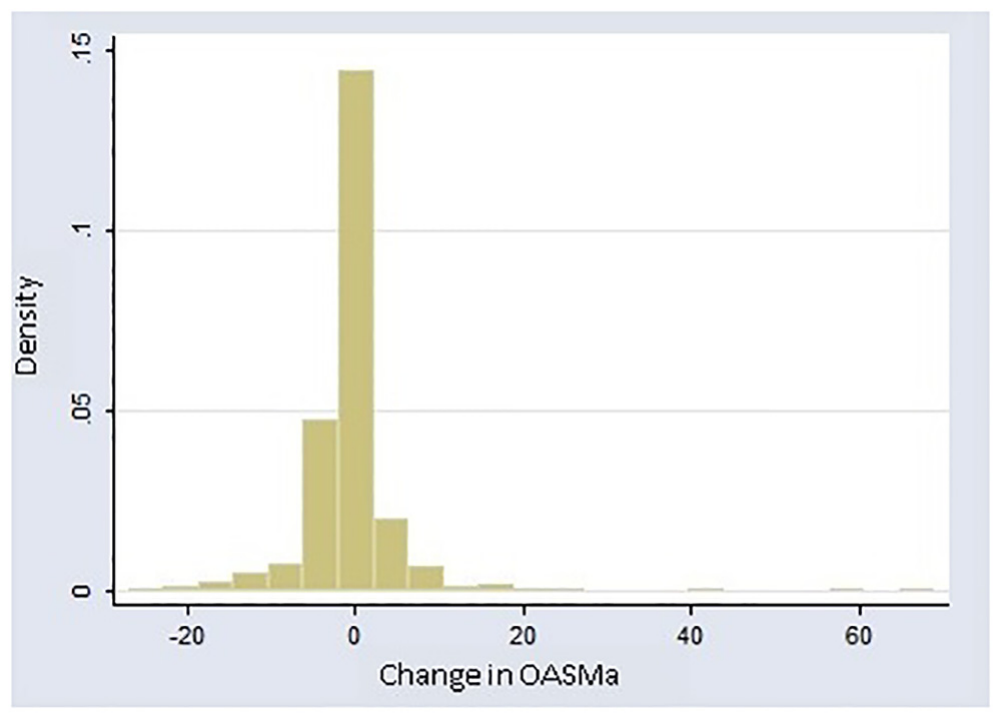
OASMa Change Values in Men. OASMa = Overt-Aggression-Scale-Modified–Aggression Subscale. Note that there are 3 values for which the absolute value of change is ≥40 that are clearly separated from the main distribution. These are the designated outliers.

Fig 1 shows the distribution of change in OASMa in men. Three extreme and influential outliers were evident on inspection of data (OASMa change ≥40 points, exceeding 6 SDs). All were large increases in aggression, all on statins—opposite to the more typical direction effect.

Tables 3–9 show results in men. Table 3 shows results of statin treatment relative to placebo in men, with and without exclusion of the influential outliers; and with and without stratification by baseline OASMa. Without excluding outliers, reduction in aggression in men was significant for pravastatin (in absence of adjustment for multiple comparisons) but not for combined statins or simvastatin. When the three outliers were excluded, significant “typical” effects were noted for statins overall, and each statin individually.

**Table 3:** Statin Effects on Aggression in Men. OASMa Change, Comparing Statin Group to Placebo.

**Retaining Outliers**												
**Baseline OASMa**	**Statins**				**Simvastatin**				**Pravastatin**			
	**N**	**Mean (SD)**	**95% CI**	**P**	**N**	**Mean (SD)**	**95% CI**	**P**	**N**	**Mean (SD)**	**95% CI**	**P**
**All**	664	-0.91 (0.50)	-1.9, 0.07	**0.070**	443	-0.79 (0.59)	-2.0, 0.37	**0.18**	441	-1.0 (0.49)	-2.0, -0.06	**0.038**
**OASMa = 0**	339	-0.43 (0.21)	-0.84, -0.02	**0.038**	226	-0.46 (0.27)	-0.99, 0.07	**0.090**	242	-0.41 (0.25)	-0.90, 0.09	**0.10**
**OASMa > 0**	325	-0.90 (1.0)	-2.9, 1.1	**0.38**	217	-0.51 (1.2)	-2.8, 1.8	**0.66**	199	-1.4 (1.0)	-3.4, 0.65	**0.18**
**Excluding Outliers (N = 3 outliers)**												
**Baseline OASMa**	**Statins**				**Simvastatin**				**Pravastatin**			
	**N**	**Mean (SD)**	**95% CI**	**P**	**N**	**Mean (SD)**	**95% CI**	**P**	**N**	**Mean (SD)**	**95% CI**	**P**
**All**	661	-1.3 (0.39)	-2.0, -0.53	**0.0009**	441	-1.4 (0.43)	-2.2, -0.53	**0.0015**	440	-1.2 (0.46)	-2.1, -0.31	**0.0083**
**OASMa = 0**	339	-0.43 (0.21)	-0.84, -0.02	**0.038**	226	-0.46 (0.27)	-0.99, 0.07	**0.090**	242	-0.41 (0.25)	-0.90, 0.09	**0.10**
**OASMa > 0**	322	-1.6 (0.76)	-3.1, -0.16	**0.030**	215	-1.6 (0.80)	-3.1, 0.03	**0.054**	198	-1.8 (0.93)	-3.6, 0.07	**0.060**

CI = confidence interval; OASMa = Overt-Aggression-Scale-Modified–Aggression-Subscale; SD = standard deviation.

**Table 4:** Sign of Aggression-Change on Statins vs Placebo in Men. (Sign of change is insensitive to outliers.)

	OR (SE)	P	95%CI
Statins N = 664	0.64 (0.097)	**0.003**	0.47, 0.86
Simvastatin N = 443	0.52 (0.094)	**<0.001**	0.37, 0.75
Pravastatin N = 441	0.77 (0.14)	**0.14**	0.54, 1.1

CI = confidence interval; OASMa = Overt-Aggression-Scale-Modified–Aggression-Subscale; OR = odds ratio from ordinal logit; SE = standard error. Analysis employs ordinal logit with robust standard errors, not adjusted for baseline OASMa. There is no exclusion of outliers, as no values represent outliers (values are collapsed to -1, 0, +1—reflecting reduction, no change, or increase in aggression, respectively). This analysis is insensitive to large magnitude outliers, as it looks only at direction (sign) not magnitude. For odds ratios derived from ordinal logit, “a unit change in the predictor variable signifies that the odds for the outcome being in a group that is greater than *k* versus less than or equal to *k* is the proportional odds times larger” [55].

**Table 5:** Men, Stratified at Age 40. (Note larger number of men in the older group.)

	*Placebo*		Statin			Simvastatin			Pravastatin		
	Mean (SD)	95%CI	Mean (SD)	95%CI	P vs placebo	Mean (SD)	95%CI	P vs placebo	Mean (SD)	95%CI	P vs placebo
**Δ OASMa Age ≤ 40** N = 92	0.036 (4.4)	-1.7, 1.8	-2.3 (5.4)	-3.6, -0.95	**0.047**	-1.7 (4.6)	-3.3, -0.11	0.14	-3.0 (6.2)	-5.4, -0.67	**0.037**
**Δ OASMa Age > 40** N = 569	0.24 (4.5)	-0.40, 0.89	-0.86 (4.6)	-1.3, -0.39	**0.007**	-1.0 (4.5)	-1.7, -0.40	**0.005**	-0.68 (4.8)	-1.4, 0.005	**0.053**
P, difference younger vs older	0.99		**0.026**			0.59			**0.020**		

CI = confidence interval; OASMa = Overt-Aggression-Scale-Modified–Aggression-Subscale; SD = standard deviation. Magnitude of effect (coefficient) is larger among men under 40, in each statin group. However, there are approximately 6 times as many participants in the over 40 vs the under 40 category, providing greater significance in the simvastatin and statin groups for those age over 40 years, despite smaller effect sizes. Last-on-treatment-value-carried-forward analysis. Excludes the 3 outliers.

**Table 6:** Double Stratification: By Age and Baseline Aggression.

**(i) Age ≤ 40**												
	**Statins (vs placebo)**				**Simvastatin (vs placebo)**				**Pravastatin (vs placebo)**			
**Stratification**	**N**	**Diff (SE)**	**95% CI**	**P**	**N**	**Diff (SE)**	**95% CI**	**P**	**N**	**Diff (SE)**	**95% CI**	**P**
“Low Aggression” OASMa = 0 at baseline (There are no outliers in this group; same with and without outliers)												
**Excluding outlier*s***	40	-2.4 (0.71)	-3.8, -0.96	**0.002**	30	-2.5 (0.89)	-4.4, -0.71	**0.008**	26	-2.2 (1.1)	-4.5, 0.09	**0.059**
**Including outliers**	40	-2.4 (0.71)	-3.8, -0.96	**0.002**	30	-2.5 (0.89)	-4.4, -0.71	**0.008**	26	-2.2 (1.1)	-4.5, 0.09	**0.059**
“High Aggression” OASMa > 0 at baseline												
**Excluding outliers**	52	-0.35 (1.9)	-4.1, 3.4	0.85	33	0.60 (1.8)	-3.0, 4.2	0.74	31	-1.4 (2.1)	-5.8, 3.0	0.52
**Including outliers**	53	1.4 (3.8)	-6.2, 9.1	0.71	34	3.9 (4.8)	-5.9, 13.6	0.42	31	-1.4 (2.1)	-5.8, 3.0	0.52
**(ii) Age > 40**												
	**Statins (vs placebo)**				**Simvastatin (vs placebo)**				**Pravastatin (vs placebo)**			
**Stratification**	**N**	**Diff (SE)**	**95% CI**	**P**	**N**	**Diff (SE)**	**95% CI**	**P**	**N**	**Diff (SE)**	**95% CI**	**P**
“Low Aggression” OASMa = 0 at baseline (There are no outliers in this group; same with and without outliers)												
**Excluding outliers**	299	-0.16 (0.21)	-0.57, 0.26	0.45	196	-0.15 (0.27)	-0.69, 0.38	0.58	216	-0.16 (0.24)	-0.64, 0.32	0.50
**Including outliers**	299	-0.16 (0.21)	-0.57, 0.26	0.45	196	-0.15 (0.27)	-0.69, 0.38	0.58	216	-0.16 (0.24)	-0.64, 0.32	0.50
“High Aggression” OASMa > 0 at baseline												
**Excluding outliers**	270	-1.8 (0.82)	-3.4, -0.15	**0.032**	182	-1.86 (0.89)	-3.6, -0.10	**0.038**	167	-1.7 (1.0)	-3.7, 0.34	0.10
**Including outliers**	272	-1.2 (1.0)	-3.2, 0.74	0.22	183	-1.3 (1.1)	-3.5, 0.89	0.24	168	-1.2 (1.1)	-3.4, 1.0	0.29

CI = confidence interval; OASMa = Overt-Aggression-Scale-Modified–Aggression-Subscale; SE = standard error. T-tests hold for the larger samples due to the Central Limit theorem, but for those with baseline values of zero, might be deemed problematic for these smaller, under 40 samples. Focusing on findings significant (or near significant) in the table above, however, ordinal logistic regression upholds the basic findings: All excluding outliers: Statin: β = -0.98 (SE = 0.40) (95%CI = -1.8, -0.19) P = 0.015. Simvastatin: β = -1.0 (SE = 0.47) (95%CI = -1.9, -0.10) P = 0.030. Pravastatin: β = -0.82 (SE = 0.43) (95%CI = -1.7, 0.02) P = 0.055. OASMa = 0: Statin: β = -1.9 (SE = 0.72) (95%CI = -3.3, -0.51) P = 0.008. Simvastatin: β = -2.1 (SE = 0.83) (95%CI = -3.7, -0.47) P = 0.01. Pravastatin: β = -1.6 (SE = 0.87) (95%CI = -3.3, 0.13) P = 0.069.

**Table 7:** Regression of Testosterone-Change on Aggression-Change in Men on Statins*.

*	Without Adjustment for LDL						Adjusted for LDL and LDL Change†					
	Change in Testosterone			Baseline Testosterone			Change in Testosterone			Baseline Testosterone		
	Beta ± SE	95% CI	P	Beta ± SE	95% CI	P	Beta ± SE	95% CI	P	Beta ± SE	95% CI	P
*On Placebo N = 162*	*0*.*059± 0*.*20*	*-0*.*33*, *0*.*45*	*0*.*77*	*-0*.*005± 0*.*21*	*-0*.*42*, *0*.*41*	*0*.*98*	*0*.*063 ± 0*.*20*	*-0*.*33*, *0*.*45*	*0*.*75*	*-0*.*015 ± 0*.*21*	*-0*.*44*, *0*.*41*	*0*.*95*
**On Statins N = 322**	0.65 ± 0.30	0.064, 1.2	**0.030**	0.83 ± 0.31	0.23, 1.4	**0.007**	0.66 ± 0.31	0.055, 1.3	**0.033**	0.85 ± 0.31	0.24, 1.5	**0.007**
**On Simvastatin N = 165**	1.3 ± 0.49	0.33, 2.2	**0.009**	1.5 ± 0.51	0.55, 2.5	**0.003**	1.3 ± 0.51	0.32, 2.4	**0.010**	1.6 ± 0.52	0.57, 2.6	**0.002**

Beta = regression coefficient; CI = confidence interval; LDL = low density lipoprotein cholesterol; OASMa = Overt-Aggression-Scale-Modified–Aggression-Subscale; SE = standard error. Change in aggression: Final on-treatment OASMa minus baseline OASMa.

* All regressions adjust for baseline aggression and baseline testosterone (as well as testosterone-change).

^†^ LDL Change is unrelated to aggression decline in these models, in which testosterone values are also adjusted: A change in testosterone (independent variable) predicts a change in aggression (dependent variable) on statins and simvastatin (of the same sign, producing a positive coefficient). The testosterone analysis was *not* robust to exclusion of influential outliers.

**Table 8:** Sleep Problems Predict Aggression in Men on Simvastatin and Combined Statins.

	Change in Sleep Problems			Baseline Sleep Problems		
	Beta ± SE	95% CI	P	Beta ± SE	95% CI	P
*On Placebo N = 188*	*0*.*39* ± *0*.*50*	*-0*.*59*, *1*.*4*	*0*.*43*	*-0*.*16* ± *0*.*12*	*-0*.*39*, *0*.*068*	*0*.*17*
**On Pravastatin N = 179**	0.21 ± 0.62	-1.0, 1.4	0.74	0.082 ± 0.13	-0.17, 0.34	0.53
**On Statins N = 363**	2.2 ± 0.55	1.1, 3.3	**<0.001**	0.23 ± 0.13	-0.035, 0.49	0.089
**On Simvastatin N = 184**	3.3 ± 0.83	1.7, 4.9	**<0.001**	0.38 ± 0.23	-0.068, 0.83	0.096

Beta = regression coefficient; CI = confidence interval; SE = standard error. Regression analysis assessing prediction of change in aggression by self-rating of change in sleep problems, stratified by treatment arm, adjusted for baseline and change in sleep problems; and baseline aggression (Overt-Aggression-Scale-Modified–Aggression-Subscale).

**Table 9:** Regression Including Both Sleep Problems and Testosterone: Both Predict Aggression in Men on Statins and Simvastatin (but Not Pravastatin or Placebo).

	Change in Testosterone			Baseline Testosterone			Change in Sleep Problems			Baseline Sleep Problems		
	Beta ± SE	95% CI	P	Beta ± SE	95% CI	P	Beta ± SE	95% CI	P	Beta ± SE	95% CI	P
*On Placebo N = 161*	*0*.*039* ± *0*.*20*	*-0*.*35*, *0*.*43*	*0*.*85*	*-0*.*033* ± *0*.*22*	*-0*.*46*, *0*.*39*	*0*.*88*	*0*.*079* ± *0*.*61*	*-1*.*1*, *1*.*3*	*0*.*90*	*-0*.*15* ± *0*.*13*	*-0*.*40*, *0*.*095*	*0*.*23*
**On Pravastatin N = 152**	-0.076 ± 0.31	-0.69, 0.54	0.81	-0.24 ± 0.32	-0.88, 0.40	0.46	0.26 ± 0.74	-1.2, 1.7	0.72	0.13 ± 0.15	-0.17, 0.44	0.39
**On Statins N = 316**	0.62 ± 0.29	0.041, 1.2	**0.036**	0.78 ± 0.30	0.19, 1.4	**0.010**	2.5 ± 0.59	1.3, 3.6	**<0.001**	0.27 ± 0.15	-0.024, 0.56	0.072
**On Simvastatin N = 164**	1.2 ± 0.47	0.31, 2.2	**0.009**	1.3 ± 0.49	0.36, 2.3	**0.007**	3.5 ± 0.84	1.8, 5.2	**<0.001**	0.28 ± 0.25	-0.21, 0.76	0.26

Beta = regression coefficient; CI = confidence interval; SE = standard error. Findings were nonsignificant for either testosterone or sleep problems (baseline or change) on pravastatin (and on placebo), which did not increase sleep problems and did not significantly reduce testosterone in this study.

### Analyses stratified by baseline aggression

Analysis stratified on baseline aggression deemed as “low baseline aggression” those men with no reported aggressive actions in the prior week (51% of the sample). None of the outliers were in this group. Separate significance was apparent in this low baseline aggression group, in whom statins use protected against later manifestations of aggression. In the “baseline aggression” group (any reported aggressive action in the prior week), significance was present with exclusion of outliers, but obviated when outliers were retained in the analysis.

The low baseline aggression group, in the “excluding outliers” comparison, had smaller effect size (in this group OASMa score cannot drop, but treatment could protect against future upward fluctuations observed on placebo), but the variance was also smaller (about 27% the effect size, 28% the SD), the latter advantaging ability to detect a difference. Retaining vs excluding outliers affected only the group with baseline aggression (reducing the effect size and increasing the variance in that group). Therefore, in analyses retaining outliers, a statin reduction in aggression in men was differentially demonstrated in the low aggression group. Despite having only around half (48%) the effect size, significance was greater in this low aggression group because the SD was proportionately lower still—the SD was less than a fourth that observed in the higher baseline aggression group (21%).

### Analysis of direction/sign of aggression-change

Because of the opposing direction sign of the outliers, obviating an otherwise significant effect, exploratory analysis was undertaken examining statin effect on *direction* of aggression-change. For this analysis, there is no need to exclude outliers as the analysis is insensitive to outliers. This affirmed that reduction in aggression was significantly more frequent in men on statins than on placebo (Table 4). Effects were separately significant for simvastatin, but not for pravastatin.

### Analysis of change-from-baseline

To further understand the findings in the face of discordant outliers, we performed an exploratory analysis capitalizing on paired t-tests to compare baseline to final aggression values, stratified by treatment assignment. (Paired t-tests assessing change from baseline often have greater power, as individuals are comparable to themselves in many respects, reducing many exogenous sources of variance. However, these inherently look *within* treatment strata, rather than comparing between them.) Among men on placebo, there was a slight rise in aggression that was not significant: 0.22 (SE = 0.30) (95%CI = -0.38, 0.82) P = 0.47. In men on statins, there was a drop in aggression, and significance was present (without excluding outliers): -0.69 (SE = 0.32) (95%CI = -1.3, -0.06) P = 0.031. Excluding outliers did not affect the placebo group, which had none of the extreme-magnitude aggression changes. Excluding the three outliers led to a larger aggression drop among men on statins: -1.1 (SE = 0.23) (95%CI = -1.5, -0.62) P<0.0001, with separate significance for simvastatin: -1.1 (SE = 0.30) (95%CI = -1.7, -0.56) P = 0.0002; and for pravastatin: -0.99 (SE = 0.34) (95%CI = -1.7, -0.32) P = 0.0040.

### Analysis stratified to consider high risk age

Men under age 40 are at greatest risk for aggression, and analyses stratified at age 40 are shown in Table 5. There were fewer male participants under age 40 than over. However effects were several times larger in younger than older men—and separately significant in this group, for combined statins and separately for pravastatin. Directly comparing men age ≤40 vs men age >40 years (OASMa change from baseline) affirms a difference by age, restricted to the statin group. On placebo, there is no suggestion of a difference: younger vs older, 0.036 (SD = 4.4) vs +.24 (SD = 4.5), P = 0.82. In contrast, the difference is significant in those on statins, younger vs older -2.3 (SD = 5.4) (95%CI = -3.6, -0.95) vs -0.86 (SD = 4.6) (95%CI = -1.3, -0.39), difference = -1.4 (SE = 0.64) (95%CI = 2.7, -0.17) P = 0.026. (Significance is separately present on pravastatin. The direction of difference was the same on simvastatin but that difference was not significant.)

Table 6 shows statin effects with double-stratification, by age and baseline aggression. The most significant reduction, that contributed strongly to the overall effect, was among men under age 40 with *low* aggression (OASMa value zero at baseline, no aggressive actions in the prior week). Men in this group could not decrease OASMa further, but were protected from upward fluctuations that might otherwise arise, more commonly in this age group. (Recall the aggression score was based on a one week slice in time; individuals who might engage in aggressive acts need not have done so in the prior week.) Among men under 40 *with* (recent) baseline aggression, the mean statin point estimate was toward *increased* aggression when outliers were included (driven by a large magnitude but nonsignificant finding with simvastatin). However, analyses without or with outliers were nonsignificant.

### Assessment for possible testosterone mediation in men

Statins are reported to affect testosterone in men [17, 19]. Lipid change on statins was previously found to significantly predict testosterone-change in men, particularly on simvastatin [17]. Change in OASMa was significantly predicted by the change in testosterone, adjusted for baseline OASMa and testosterone in men (Table 7). This was true for men on statins, and particularly on simvastatin. Baseline testosterone was also a significant predictor. Including LDL (change and baseline) in the model, the testosterone relationships were preserved (with LDL relationships nonsignificant). However, the testosterone finding, though significant, was not robust and depended on inclusion of an influential outlier.

### Assessment for potential mediation by change in sleep problems

Sleep problems were previously shown to increase significantly on simvastatin but not pravastatin [20], and sleep problems are linked to aggression [21]. Table 8 shows results of regression analyses assessing whether change in sleep problems may contribute as a mediating factor in change in aggression, stratified by treatment arm. Change in sleep problems on combined statins, and simvastatin separately—but not on pravastatin or placebo—was a highly significant predictor of change in aggression in men.

### Assessment for potential mediation by change in testosterone and in sleep problems: an explanation for bidirectional effects

**Fig 2:**
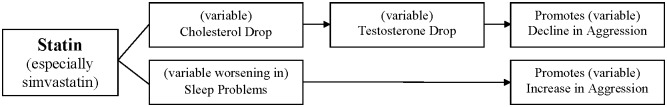
Typical Statin Effects on Testosterone (Decrease) and on Sleep Problems (Increase) Influence Aggression in Opposite Directions. LDL = low density lipoprotein cholesterol.

Table 9 shows results of regression analyses including both testosterone and sleep problems (baseline and change), stratified by treatment assignment. Again, only simvastatin significantly increased sleep problems in this sample, and simvastatin significantly reduced testosterone. On combined statins, and on simvastatin separately (but not on placebo or pravastatin), both change in testosterone and change in sleep problems were significant predictors of change in aggression. In both cases the sign of the coefficient for the predictor was positive, but since simvastatin affected these predictors in opposite directions, they help to account for effects on aggression in both directions (Fig 2). The positive relationships mean those with a (greater) drop in testosterone on simvastatin experience a (greater) expected *decline* in aggression; while those manifesting a (greater) increase in sleep problems on simvastatin will have a (greater) expected *increase* in aggression. This supports the presence of bidirectional mechanisms of simvastatin on aggression, in men, and helps to account for the outliers. (The two most extreme outliers, with OASMa increases of 57 and 69, were both on simvastatin; and both rated change from baseline in sleep problems as “much worse” on simvastatin.)

### Women

Women comprised ~1/3 of the sample (i.e. approximately half as many women as men), as expected due to eligibility restriction to surgically or chronologically postmenopausal women. Women were separately randomized.

There was a trend to lower baseline OASMa in the placebo group. Among women who provided follow-up values, baseline OASMa was lower among women on placebo (1.5±2.8) than on statins (2.6±4.3, P = 0.024), necessitating use of regression to adjust for baseline disparities, in analyses for women.

Table 10 shows effects of statins on OASMa in women. Based on regression adjusted for baseline OASMa, statin use was associated with a trend to increased aggression (change) on statins in women, in the full sample. Findings were significant excluding those with early or surgical menopause (age<45), totaling seven participants, yielding N = 304: β = 0.68 (SE = -0.34) (95%CI = 0.005, 1.4) P = 0.048. {In fact, on inspection, a single, surgically menopausal woman of age 44—surgery at age 36—produced the loss of significance in the full sample. Excluding this woman led the statin increase in aggression to be significant.}

**Table 10:** Statin Effects on Aggression in Women.

	Statin				Simvastatin				Pravastatin			
	N	Beta (SE)	95% CI	P vs placebo	N	Beta (SE)	95% CI	P vs placebo	N	Beta (SE)	95% CI	P vs placebo
**ΔOASMa, all women**	311	*0*.*59 (0*.*35)*	*-0*.*11*, *1*.*3*	*0*.*098*	*207*	*0*.*87 (0*.*51)*	*-0*.*14*, *1*.*9*	*0*.*092*	*208*	*0*.*29 (0*.*38)*	*-0*.*46*, *1*.*0*	*0*.*45*
**ΔOASMa Excluding one participant 412**	310	0.70 (0.34)	0.035, 1.4	**0.039**	206	0.98 (0.50)	-0.010, 2.0	**0.052**	207	0.40 (0.36)	-0.32, 1.1	0.27
**ΔOASMa** all **Age ≥45**	304	0.68 (0.34)	0.0053, 1.4	**0.048**	202	0.95 (0.51)	-0.054, 2.0	**0.063**	203	0.39 (0.37)	-0.34, 1.1	0.30
**ΔOASMa** all **Age ≥ 50**	288	0.71 (0.35)	0.018, 1.4	**0.044**	194	1.1 (0.54)	0.017, 2.2	**0.046**	194	0.33 (0.37)	-0.40, 1.0	0.37
**ΔOASMa** all **Age ≥ 55**	238	0.77 (0.39)	0.0040, 1.5	**0.049**	158	1.3 (0.61)	0.11, 2.5	**0.033**	160	0.24 (0.38)	-0.50, 0.98	0.52

Beta = regression coefficient; CI = confidence interval; OASMa = Overt-Aggression-Scale-Modified–Aggression-Subscale; SE = standard error. A statin x age interaction term, e.g. binarizing at age 55 (years) was significant for simvastatin (P = 0.039), and borderline significant for combined statins (P = 0.092). Women age <45 years, including participant 412: Statin: n = 7; β = -0.79 (SE = 2.5) 95%CI = -7.9, 6.3; P = 0.77. Simvastatin: n = 5; β = 0.15 (SE = 4.8) 95%CI = -20.5, 20.8; P = 0.98. Pravastatin: n = 5; β = -1.2 (SE = 3.3) 95%CI = -15.3, 12.9; P = 0.74. Women age <45 years, excluding participant 412: Statin: n = 6; β = 3.5 (SE = 1.8) 95%CI = -2.2, 9.2; P = 0.15. Simvastatin: n = 4; β = 5.0 (SE = 1.4) 95%CI = -13.0, 23.0; P = 0.18. Pravastatin: n = 4; β = 2.0 (SE = 1.4) 95%CI = -16.0, 20.0; P = 0.39.

The increase in aggression in women in a more typical postmenopausal age group, age ≥45, was not highly sensitive to cutoff age: confining analysis to age ≥50 retained significance for statins vs placebo and added separate significance for simvastatin, and significance persisted with restriction to women age ≥55 despite further attrition of sample size.

Table 11 shows analyses stratified by baseline aggression in women. A significant aggression increase on statins was apparent among women without evidence of baseline aggression. In this group, pravastatin contributed more strongly than simvastatin. The group without baseline aggression was free of baseline disparities in aggression that affected women overall, and included no aggression-change outliers. Women within this group appeared well matched across randomization arms. Among women with any recent aggression at baseline, no significant statin effect was observed: tendencies were toward a reduction in aggression on pravastatin, and an increase on simvastatin (rising with age). The absolute value of the effect sizes on simvastatin and pravastatin were not grossly dissimilar to the effect sizes that was significant in women without baseline aggression, but standard errors were substantially higher and findings did not approach significance.

**Table 11:** Women Stratified by Baseline Aggression Score.

**(i) Women**, **Low Baseline Aggression (OASMa = 0 at baseline**, **no aggressive actions in the prior week)**.												
	**Statins (vs placebo)**				**Pravastatin (vs placebo)**				**Simvastatin (vs placebo)**			
**Age**	**N**	**Beta** (SE)	95% CI	**P**	**N**	**Beta** (SE)	95% CI	**P**	**N**	**Beta** (SE)	95% CI	**P**
**All Ages**	175	**0.84** (0.30)	0.24,1.4	**0.006**	122	**1.02** (0.43)	0.16, 1.9	**0.020**	118	**0.65** (0.38)	-0.10, 1.4	**0.09**
**≥ 45**	171	**0.80** (0.30)	0.20, 1.4	**0.009**	119	(0.44)	0.14, 1.9	**0.023**	115	**0.58** (0.37)	-0.16, 1.3	**0.12**
**≥ 50**	163	**0.75** (0.30)	0.16, 1.3	**0.013**	115	**0.85** (0.41)	0.04, 1.7	**0.041**	111	**0.66** (0.40)	-0.13, 1.4	**0.10**
**≥ 55**	137	**0.82** (0.32)	0.20, 1.4	**0.010**	96	(0.46)	0.11, 1.9	**0.028**	92	**0.61** (0.40)	-0.19, 1.4	**0.13**
**(ii) Women**, **With Baseline Aggression (OASMa >0 at baseline**, **≥1 aggressive action(s) in the prior week)**.												
	**Statins (vs placebo)**				**Pravastatin (vs placebo)**				**Simvastatin (vs placebo)**			
**Age**	**N**	**Beta** (SE)	95% CI	**P**	**N**	**Beta** (SE)	95% CI	**P**	**N**	**Beta** (SE)	95% CI	**P**
**All Ages**	136	**0.071** (0.73)	-1.4, 1.5	0.92	86	**-0.81** (0.69)	-2.2, 0.55	0.24	89	**0.89** (1.0)	-1.1, 2.9	0.38
**All except participant 412**	135	**0.34** (0.68)	-1.0, 1.7	0.61	85	**-0.54** (0.64)	-1.8, 0.73	0.40	88	**1.2** (0.98)	-0.78, 3.1	0.24
**≥ 45**	133	**0.37** (0.69)	-0.99, 1.7	0.59	84	**-0.53** (0.64)	-1.8, 0.75	0.41	87	**1.2** (1.0)	-0.78, 3.2	0.23
**≥ 50**	125	**0.48** (0.72)	-0.94, 1.9	0.50	79	**-0.48** (0.68)	-1.8, 0.86	0.48	83	**1.4** (1.0)	-0.73, 3.4	0.20
**≥ 55**	101	**0.48** (0.81)	-1.1, 2.1	0.56	64	**-0.95** (0.68)	-2.3, 0.40	0.17	66	**1.9** (1.2)	-0.54, 4.3	0.13

Regression with robust SE, adjusted for baseline OASMa. Beta = regression coefficient; CI = confidence interval; OASMa = Overt-Aggression-Scale-Modified–Aggression-Subscale; SE = standard error. (ii) Simvastatin, in age >55, shows a significant treatment-by-baseline aggression interaction, P = 0.043. Reduction trend for pravastatin, and increase trend for simvastatin is similar to effects observed in men under age 40 with baseline aggression. Of note, estimated absolute effect magnitudes among those *with* baseline aggression (reduced aggression for pravastatin and increased aggression for simvastatin), though nonsignificant, are comparable to effect magnitudes that were significant in those *without* baseline aggression; however, Ns are smaller, and SEs for aggression changes are materially higher.

For age ≥55, there was a significant interaction effect of simvastatin with baseline presence/absence of aggression: β = 1.2 (SE = 0.50) (95%CI = 0.22, 2.2) P = 0.017, i.e. simvastatin increased aggression more strongly in those with aggression >0 at baseline.

In contrast to men, no significant relationship between testosterone or sleep change and aggression-change was noted in women—though, power to see such relationships was also reduced (data not shown).

### Serotonin findings

The cross-sectional relationship between whole blood serotonin and aggression previously reported in young men [26] was affirmed in this sample: men age ≤40: correlation coefficient 0.27 (P = 0.008). It was affirmed this did not apply to young women, for whom nonsignificant correlation bore opposite sign (young defined for women as under 50, as there were no women under 40): correlationcoefficient -0.12 (P = 0.58). We newly show that it also did not apply for older men, e.g. age >55 correlation coefficient 0.0046, P = 0.93.

Statins did not affect whole blood serotonin in the full sample, or in men or women as a whole, nor in young men in whom serotonin related to aggression. Exploratory analysis suggested an effect of statins on whole blood serotonin in older women, strengthening with age, and significant in women age >60 (if adjustment for baseline serotonin was included, via regression): *Statin* (N = 156): β = -158 (SE = 71.6) (95%CI = -299, -16.2) P = 0.029; *Simvastatin* (N = 107): β = -139 (SE = 82.3) (95%CI = -302, 24.1) P = 0.094; *Pravastatin* (N = 102): β = -181 (SE = 77.1) (95%CI = -334, -27.8) P = 0.021. As for many findings, significance was strengthened with completer analysis: *Statins* (N = 147): β = -184 (SE = 76.0) (95%CI = -333, -34.6) P = 0.016; *Simvastatin* (N = 99): β = -173 (SE = 87.4) (95%CI = -346, 0.88) P = 0.051; *Pravastatin* (N = 96): β = -202 (SE = 80.5) (95%CI = -361, -42.1) P = 0.01. However, there was no suggestion of a relationship between change in whole blood serotonin and change in aggression on statins, or on either statin separately, in this group of women overall (women overall: β = 0.00046 (SE = 0.00045) (95%CI = -0.00042, 0.0013) P = 0.30).

## Discussion

### Recap of findings

Statins showed a significant sex-interaction, with opposite direction point estimates for statin differences from placebo in on-treatment OASMa in men and women. Trends in men were toward a reduction in aggression on statins. The reduction in aggression was strongly significant if three influential outliers were excluded. These three represented the participants with the largest changes in aggression—many times the SD, in each case an increase, in each case on statins, effectively serving as counterweights to the typical finding of aggression reduction. The reduction was significant when direction of effect was examined, without excluding outliers (this analysis is insensitive to outliers). And the reduction was significant comparing on-treatment to baseline values, in each statin group (but not on placebo). Among men, statins conferred significant protection from aggression relative to placebo in those with “low” baseline aggression in younger age (no reported aggressive actions in the prior week), but not those with recent aggression at baseline. Effects might be more uniform, and lower variance might facilitate detection of effects, in those with low aggression at baseline. Alternatively an explanation considering statin prooxidant/antioxidant duality and cell energetics could also readily explain greater benefit in those with no aggression at baseline, and more mixed effects of statins in those with baseline manifestations of aggression. Aggression has been linked to numerous states involving low physiological energy supply [56–61], which may be more vulnerable to prooxidant and associated energy-adverse effects of statins [50].

A significant age-statin interaction was present, and younger men, under age 40, though few in number, showed a large and independently significant reduction in aggression, again more pronounced in those with low baseline aggression (no recent aggressive behaviors, at the baseline visit).

Both sleep problems and drop in testosterone (men) have been reported on statins, particularly on simvastatin, relative to placebo. In men, change/reduction in aggression on statins related significantly to change/reduction in testosterone on statins. (This was, however, sensitive to exclusion of outliers.) Additionally, change/increase in aggression on statins related significantly to change/increase in sleep problems on statins, driven by effects on simvastatin. Relations of testosterone-change and change in sleep problems to aggression in men were significant whether assessed separately, or together. This offered a potential foundation for the several striking counter-directional outliers observed in men: the two most extreme outliers had been randomized to simvastatin, and both developed “much worse” sleep problems on simvastatin. Thus, sleep change and testosterone change represent identified potential mediators that may help to explain both the typical direction effect in men, and the exceptions. The possibility that other mediators and effect modifiers may influence effects of statins on aggression cannot be excluded.

For (postmenopausal) women, statins tended to increase aggression, an effect that was significant with exclusion of one surgically menopausal woman; or all age <45 (with early or surgical menopause). As was the case for *reductions* in aggression in men, *increases* in aggression in women were most evident among those with low baseline aggression. Those with evidence of aggression at baseline have other forces acting on aggression, and may be subject to more variance in aggression arising from sources distinct from statins. Resulting added variance may reduce ability to detect the statin effect—whether the typical rise in aggression in women, or the typical fall in aggression in men—among those with baseline evidence of aggression. Additionally, it is possible that statins may interact, in potentially different ways, with the factors that underlie the aggression, in both sexes.

### Fit with existing literature

These findings provide the first RCT evidence relevant to understanding statin effects on aggression. Findings substantially comport with available literature on statins and aggression. Meta-analysis of statin trials (predominating at the time in nonelderly men) have not shown an increase in violent deaths [13]; any trend was toward reduction, consistent with effects observed in men and younger age here. Changes in testosterone on statins, tied to LDL reductions on simvastatin, *might* help to mediate aggression changes in men, but the testosterone effect, though significant, was not robust. Outliers in this study (large aggression increases, exclusively observed on statins), may plausibly map to individuals with (atypical) sizeable increases in irritability/ aggression, reported in the literature [14, 15]. Our findings of increased aggression in women (age ≥45) is consistent with greater aggression in women on lipid-lowering medications in the WISE study [16]. The mean age of 62 years in that study matches the mean age of our age≥45 females (mean age 62.2 years), in whom a significant aggression increase on statins was observed.

The opposing direction effects in men and women may seem counterintuitive. In fact, however, bidirectional effects of statins for many outcomes have been reported, such as for glucose, proteinuria, cancer, and possibly cognition, with participant characteristics modifying relative likelihood of favorable or adverse effects [50]. Both older age and female sex have predicted less favorable statin effects for many outcomes (extending to all-cause mortality) [50, 62], and appear to do so here (counting lower aggression as more favorable). We speculate that antioxidant-prooxidant duality of statin effects, and effects on cell energy balance may play a role here, as has been postulated for other outcomes [50]. Those in whom mitochondrial insufficiency contributes to baseline aggression might be at risk for increased problems on statins (as diminutions in, say, coQ10 production and transport unmask mitochondrial dysfunction [63] and increased free radical release [64, 65]); while behavior in those with inflammation and oxidative stress *not* arising in settings of mitochondrial compromise (i.e. occurring in settings in which statin antioxidant effects may commonly predominate) might benefit from statin-induced benefits to inflammation, oxidative stress and blood flow. Provisional support for a role of such factors is suggested by a significant positive relationship between aggression and muscle weakness within this study sample (P<0.0001), coupled with known relationships of muscle symptoms to statin effects on mitochondria and oxidative stress [50, 66, 67]. Additional research is required to better understand effect modification within male and female groups with higher baseline aggression.

### Limitations

Characteristics of participating men and women differed, consistent with different characteristics of statin using men and women. Though it was prespecified that a significant sex-interaction would lead to sex-stratified analysis, power calculations were not based on the presumption of sex stratification, with different effects in each group. Nonetheless, important differences were evident for men and women, and findings in both sexes suggesting differential effects related to baseline aggression (or at least, differential effect size relative to variance) provide key data to inform design of future studies in this area. Their potential teratogenicity means statins should be used with caution in women of procreative potential, and risk of cardiac disease in women lags that in men, so that female statin users in the real world, as in this study, are typically older. The study sampled broadly, to relatively reflect the range of statin users whom it would be deemed acceptable to randomize. This reduces sample homogeneity and adds variance—potentially attenuating ability to identify effects that may be present in a more homogeneous subgroup. However, broad sampling does enable important comparative assessments, such as by age, and by baseline aggression. Testosterone assessment involved total testosterone; free testosterone was not assessed. Additionally, whole blood serotonin was used as a proxy, central measures of serotonin, such as cerebrospinal fluid 5-hydroxyindoleacetic acid (CSF 5HIAA), were not procured.

Our analysis approach was refined following a change in statistician between when the study was proposed and when analyses took place. Multiple comparison adjustments are grounded in the presumption that the first order explanation for findings is chance [68]. In hypothesis driven research where there are reasons that variables may relate, and in this case particularly in the setting of the testosterone and sleep findings (bearing triangulating evidence, with relationships to known predictor variables affirmed), chance is no longer the first order explanation. The primary finding in men (combined statins vs placebo, in the analysis excluding outliers) would, however, retain significance even with multiple comparison adjustment. Additional analyses, serving to buttress the findings and to understand them, are not independent, nor subjected to multiple comparison adjustment. The sample size for women is half that for men, calculations did not power separately for women, and significance of findings for women would not be sustained under multiple comparison adjustment. However, the significance of the sex-interaction term, the absence of a testosterone mechanism for aggression reduction in women, as well as evidence that other outcomes that bear a relationship to oxidation-anti-oxidation and cell energy (as does aggression) have been less favorable on statins in women and older age (women were also older), enhance confidence in the findings.

### Implications

Statins typically reduced aggression in men, particularly younger men (there was a significant age interaction); and typically increased aggression in postmenopausal women. These effects were most consistent and most significant in those with low baseline aggression, likely in part owing to lesser influence by other sources of fluctuation in aggression.

## Supporting Information

S1 Appendix Appendix of supporting information files. (PDF)

S1 CONSORT Checklist CONSORT checklist. (DOC)

S1 Fig Abbreviated Mevalonate Pathway. Depicts site of inhibition by statins; shows cholesterol and testosterone as products of the affected pathway.
(TIF)

S2 Fig Consort Flow Diagram: Combined Sample (Men & Women). Reasons for participant drops are outlined in S1 Table.
(TIF)

S3 Fig Consort Flow Diagram: Men. Reasons for participant drops are outlined in S1 Table.
(TIF)

S4 Fig Consort Flow Diagram: Women. Reasons for participant drops are outlined in S1 Table.
(TIF)

S1 Table Reasons for study drops. (DOC)

S2 Table Statin effects on lipids. (DOC)
