# Supplementary material for: Statin Effects on Aggression: Results from the UCSD Statin Study, a Randomized Control Trial
Source: PLoS One. 2015 Jul 1;10(7):e0124451. doi: 10.1371/journal.pone.0124451 (PMC4488854; doi:10.1371/journal.pone.0124451)
Supplement: S1 Appendix — (PDF) [file pone.0124451.s001.pdf]

Appendix I

Figure 1. Abbreviated Mevalonate Pathway...……………………………………………...Page 2

Figure 2. Consort Flow Diagram: Combined Sample (Men & Women)...…………………Page 3

Figure 3. Consort Flow Diagram: Men…………………………...……………...…Page 4

Figure 4. Consort Flow Diagram: Women…………………………...……………….....Page 5

Table 1. Reasons for Study Drops……………………………...…………………...Page 6

Table 2. Statin Effects on Lipids………………………………...…………………….Page 7

Figure 1. Abbreviated Mevalonate Pathway.

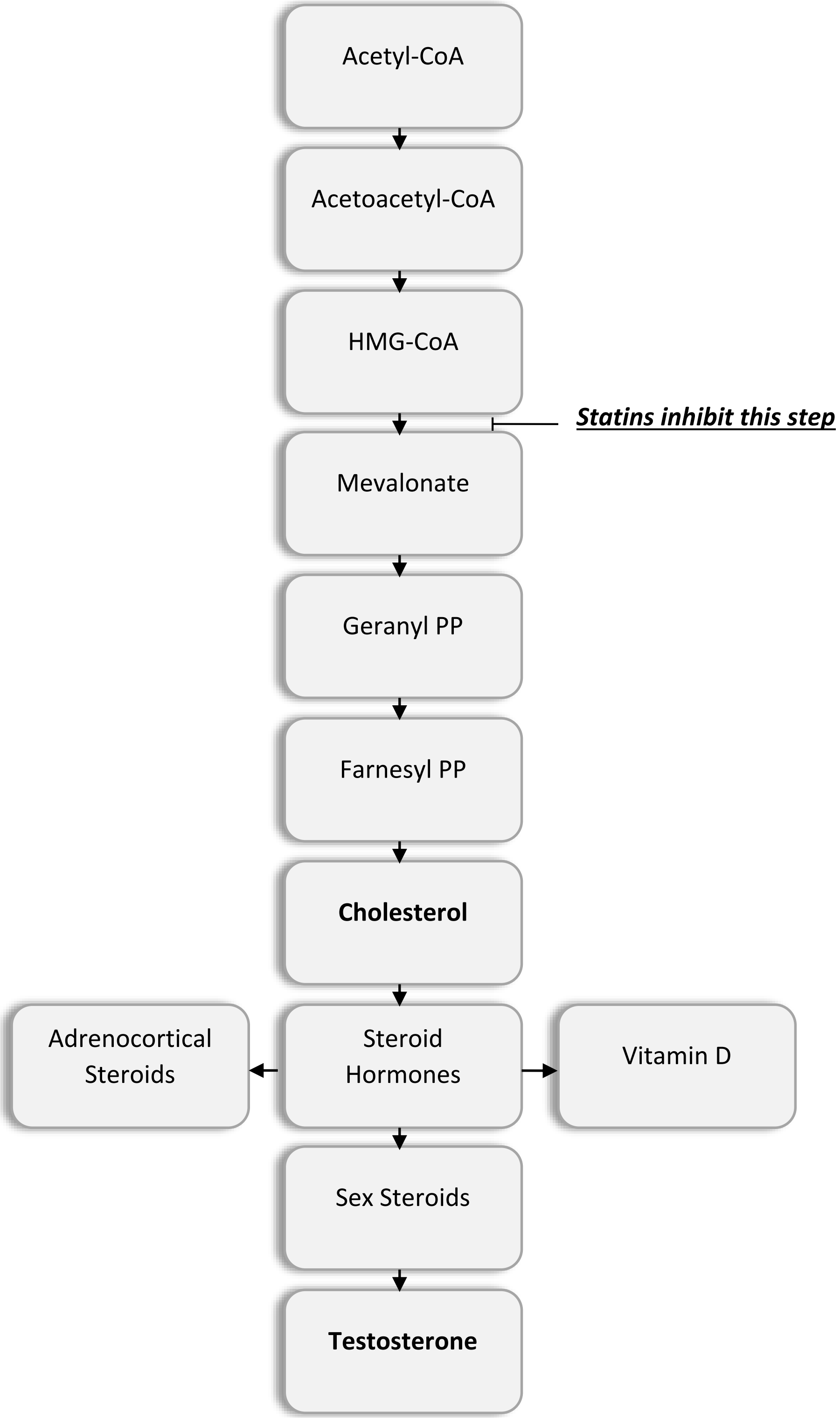

Figure 1 legend. Depicts site of inhibition by statins; shows cholesterol and testosterone as products of the affected pathway.

Figure 2. Consort Flow Diagram: Combined Sample (Men & Women).

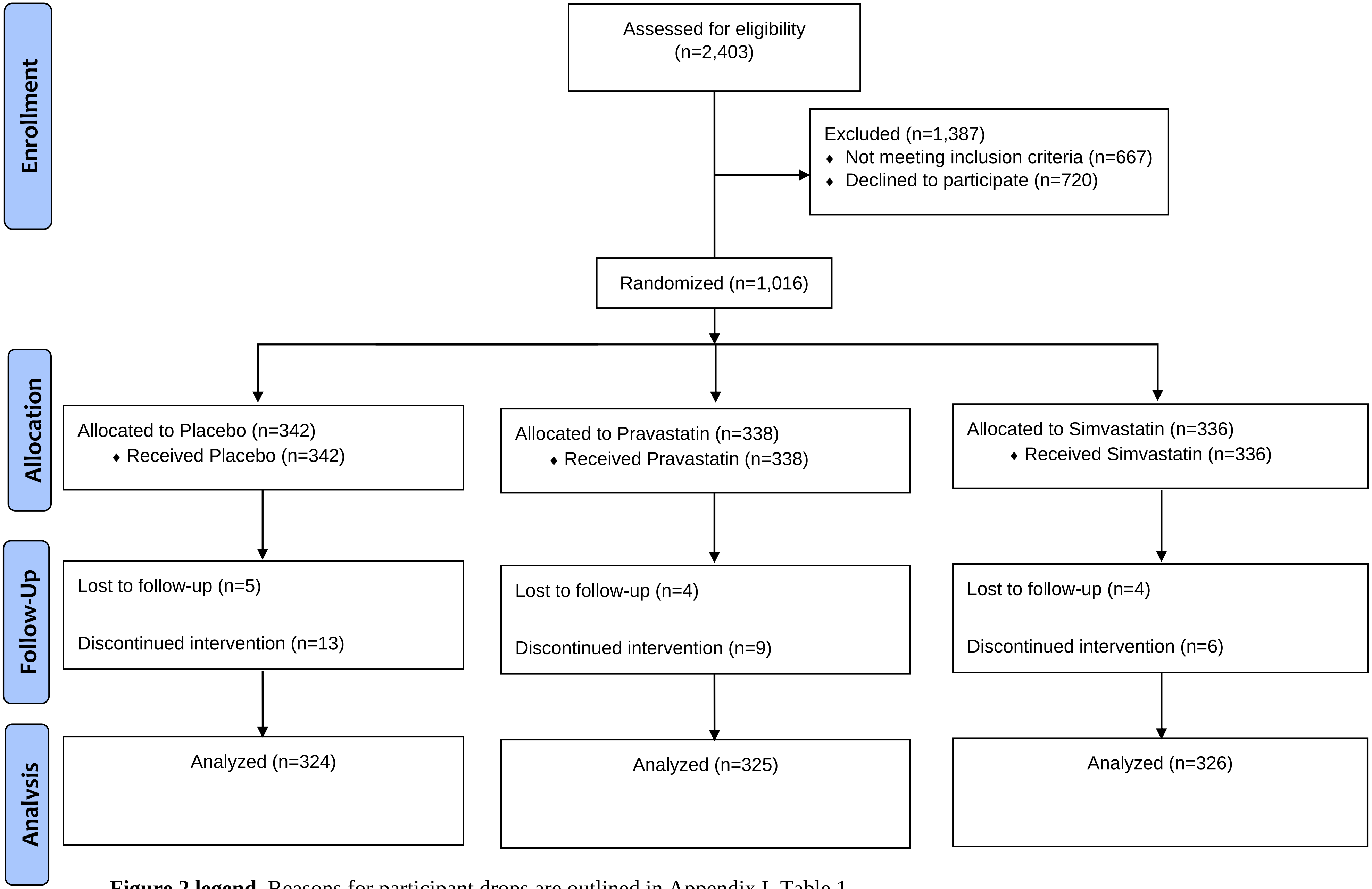

Figure 2 legend. Reasons for participant drops are outlined in Appendix I, Table 1.

Figure 3. Consort Flow Diagram: Men.

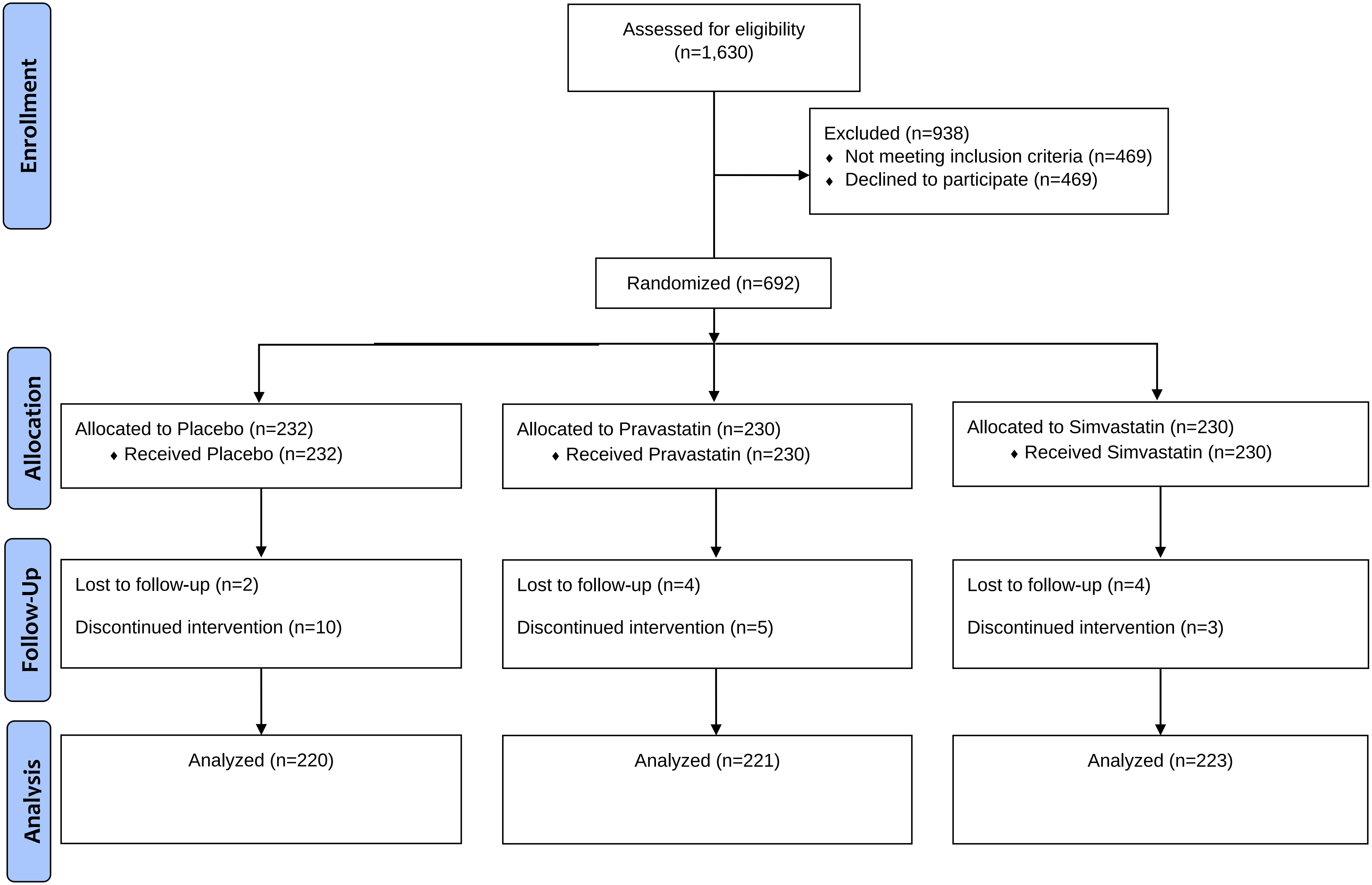

Figure 3 legend. Reasons for participant drops are outlined in Appendix I, Table 1.

Figure 4. Consort Flow Diagram: Women.

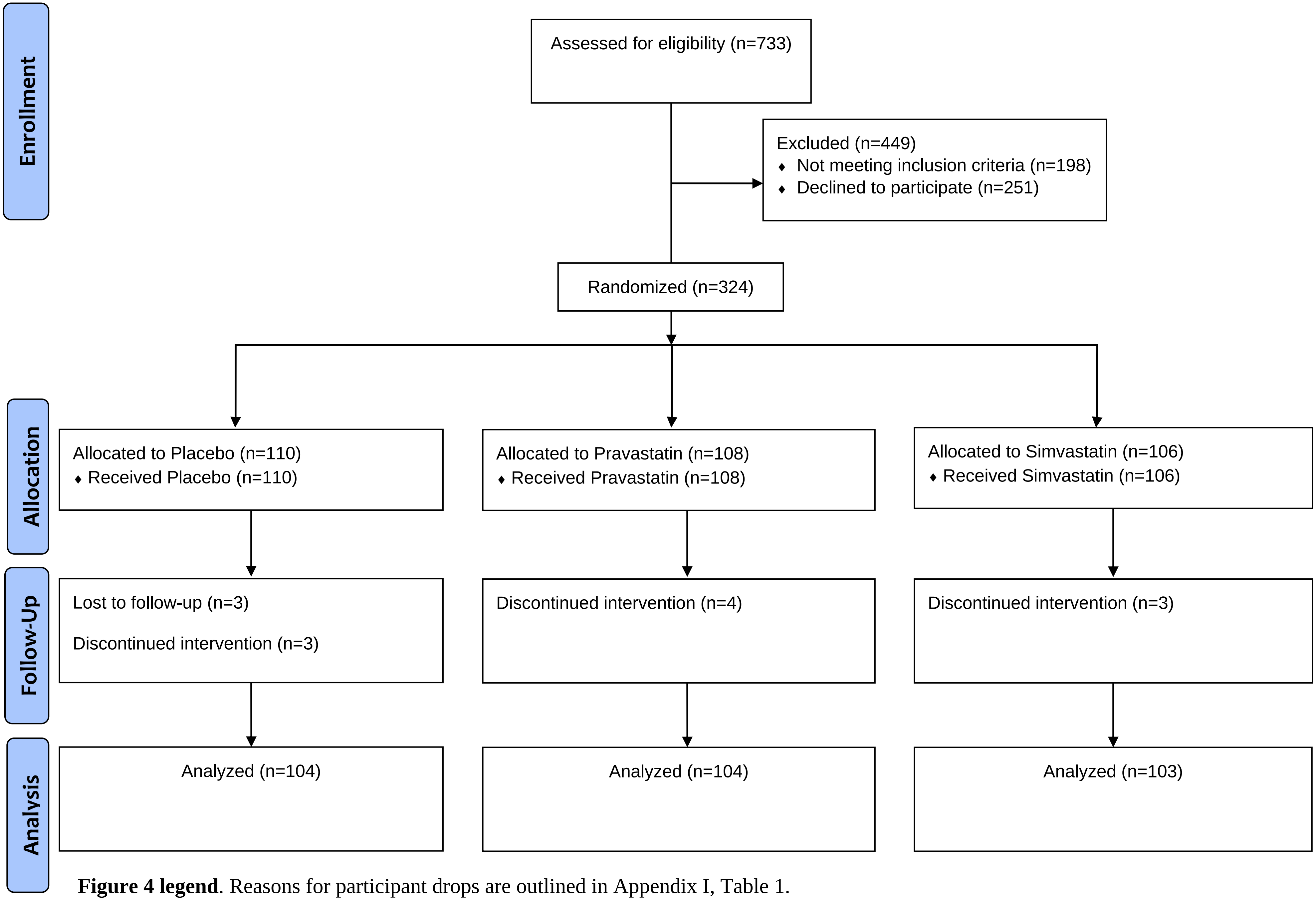

Figure 4 legend. Reasons for participant drops are outlined in Appendix I, Table 1.

Table 1. Reasons for Study Drops.

	Placebo	Pravastatin	Simvastatin
Scheduling	4	1	0
Disliked aspect of study visit (e.g. too far, disliked blood draw, questionnaires, etc)	2	1	0
Physician/PCP recommended they not participate	2	0	2
Surgery (or issues related to medical condition predating participation)	3	1	2
Symptoms	2<br><br>a) Felt dizzy<br><br>b) Felt uneasiness (which dissipated) and some muscular pain	4<br><br>a) Irritability, depression, suicidal thoughts and nausea after 10 pills<br><br>b) Leg pain that subsided following discontinuation<br><br>c) Muscle pain attributed to study medication<br><br>d) Upper respiratory infection	1<br><br>Muscle pain (also concerned about alcohol plus study medication)
Miscellaneous	0	2<br><br>a) Went out of town, forgot to take meds, did not return for f/u<br><br>b) Due to preexisting tracheostomy, could not take study medications without opening capsules	1<br><br>Study psychiatrist felt participation inappropriate (history of depression and suicide attempt)

Table 2. Statin Effects on Lipids.

	All				Men				Women			
	Mean (SD)<br>95%CI				Mean (SD)<br>95%CI				Mean (SD)<br>95%CI			
**Completers**	N=279	N=558	N=280	N=278	N=193	N=368	N=184	N=184	N=86	N=190	N=96	N=94
	**Placebo**	**Statin**	**Prava**	**Simva**	**Placebo**	**Statin**	**Prava**	**Simva**	**Placebo**	**Statin**	**Prava**	**Simva**
ΔTotal cholesterol, completer	-2.10<br>(25.8)<br>-92, 86	-51.9<br>(29.1)<br>-134, 69	-46.6<br>(28.5)<br>-102, 42	-57.2<br>(28.7)<br>-134, 69	-3.01<br>(24.7)<br>-92, 57	-51.1<br>(27.7)<br>-124, 42	-46.8<br>(28.9)<br>-102, 42	-55.3<br>(25.9)<br>-124, 18	-0.058<br>(28.0)<br>-76, 86	-53.6<br>(31.5)<br>-134, 69	-46.3<br>(27.9)<br>-101, 30	-61.0<br>(33.4)<br>-134, 69
P	---------	**<0.0001**	**<0.0001**	**<0.0001**	---------	**<0.0001**	**<0.0001**	**<0.0001**	---------	**<0.0001**	**<0.0001**	**<0.0001**
ΔLDL, completer	-1.10<br>(24.0)<br>-84, 67	-44.7<br>(25.1)<br>-103, 78	-40.4<br>(24.4)<br>-103, 28	-49.0<br>(25.1)<br>-102, 78	-1.96<br>(23.4)<br>-84, 67	-44.4<br>(23.8)<br>-102, 34	-41.0<br>(24.2)<br>-95, 28	-47.7<br>(22.9)<br>-102, 34	0.83<br>(25.4)<br>-71, 60	-45.4<br>(27.6)<br>-103, 78	-39.4<br>(24.9)<br>-103, 27	-51.6<br>(29.0)<br>-95, 78
P	---------	**<0.0001**	**<0.0001**	**<0.0001**	---------	**<0.0001**	**<0.0001**	**<0.0001**	---------	**<0.0001**	**<0.0001**	**<0.0001**
ΔHDL, completer	-1.80<br>(8.71)<br>-34, 33	-1.89<br>(9.69)<br>-37, 47	-1.85<br>(9.98)<br>-36, 47	-1.94<br>(9.40)<br>-37, 32	-1.08<br>(8.51)<br>-34, 33	-1.23<br>(8.65)<br>-37, 45	-1.74<br>(8.72)<br>-36, 45	-0.72<br>(8.58)<br>-37, 32	-3.41<br>(9.01)<br>-26, 19	-3.16<br>(11.3)<br>-36, 47	-2.04<br>(12.1)<br>-36, 47	-4.31<br>(10.5)<br>-32, 18
P	---------	**0.89**	**0.95**	**0.86**	---------	**0.84**	**0.45**	**0.69**	---------	**0.86**	**0.39**	**0.54**
ΔTrig, completer	-2.09<br>(58.9)<br>-333, 275	-19.5<br>(60.5)<br>-623,285	-13.1<br>(58.3)<br>-266,285	-25.9<br>(62.0)<br>-623, 181	-2.11<br>(64.1)<br>-333, 275	-17.6<br>(61.0)<br>-266, 285	-9.78<br>(65.4)<br>-266,285	-25.3<br>(55.2)<br>-192, 181	-2.03<br>(45.1)<br>-118, 118	-23.2<br>(59.5)<br>-623, 72	-19.3<br>(41.0)<br>-151, 67	-27.1<br>(73.8)<br>-623, 72
P	---------	**0.0001**	**0.027**	**<0.0001**	---------	**0.0053**	**0.25**	**0.0002**	---------	**0.0036**	**0.0073**	**0.0072**
**LVCF**	**N=322**	**N=645**	**N=324**	**N=321**	**N=219**	**N=440**	**N=220**	**N=220**	**N=103**	**N=205**	**N=104**	**N=101**
ΔTotal cholesterol, LVCF	-1.69<br>(25.2)<br>-92, 86	-51.2<br>(28.6)<br>-134, 69	-46.2<br>(28.0)<br>-102, 42	-56.3<br>(28.4)<br>-134, 69	-2.43<br>(24.2)<br>-92, 57	-50.4<br>(27.4)<br>-124, 42	-46.4<br>(28.3)<br>-102, 42	-54.4<br>(25.9)<br>-124, 28	-0.11<br>(27.4)<br>-76, 86	-53.1<br>(31.1)<br>-134, 69	-45.8<br>(27.5)<br>-101, 30	-60.6<br>(32.9)<br>-134, 69
P	---------	**<0.0001**	**<0.0001**	**<0.0001**	---------	**<0.0001**	**<0.0001**	**<0.0001**	---------	**<0.0001**	**<0.0001**	**<0.0001**
ΔLDL, LVCF	-0.98<br>(24.0)<br>-84, 67	-44.5<br>(24.9)<br>-105, 78	-40.2<br>(24.6)<br>-105, 28	-48.9<br>(24.6)<br>-102, 78	-1.24<br>(22.8)<br>-84, 67	-44.1<br>(23.8)<br>-105, 34	-40.5<br>(24.6)<br>-105, 28	-47.6<br>(22.5)<br>-102, 34	-0.44<br>(26.4)<br>-71, 65	-45.5<br>(27.2)<br>-103, 78	-39.4<br>(24.6)<br>-103, 27	-51.8<br>(28.5)<br>-95, 78
P	---------	**<0.0001**	**<0.0001**	**<0.0001**	---------	**<0.0001**	**<0.0001**	**<0.0001**	---------	**<0.0001**	**<0.0001**	**<0.0001**
ΔHDL, LVCF	-1.51<br>(8.87)<br>-34, 33	-1.46<br>(9.60)<br>-37, 47	-1.51<br>(9.95)<br>-36, 47	-1.40<br>(9.26)<br>-37, 32	-1.08<br>(8.74)<br>-34, 33	-0.92<br>(8.53)<br>-37, 45	-1.56<br>(8.55)<br>-36, 45	-0.28<br>(8.48)<br>-37, 32	-2.42<br>(9.11)<br>-26, 21	-2.60<br>(11.5)<br>-36, 47	-1.40<br>(12.5)<br>-36, 47	-3.84<br>(10.4) -<br>32, 18
P	---------	**0.94**	**0.99**	**0.88**	---------	**0.83**	**0.56**	**0.33**	---------	**0.89**	**0.50**	**0.30**
ΔTrig, LVCF	-1.84<br>(58.6)<br>-333, 275	-19.0<br>(61.3)<br>-623,285	-13.4<br>(60.1)<br>-266,285	-24.6<br>(62.0)<br>-623, 181	-2.27<br>(62.0)<br>-333, 275	-16.9<br>(62.2)<br>-266, 285	-10.9<br>(67.3)<br>-266,285	-22.9<br>(56.0)<br>-192, 181	-0.92<br>(51.1)<br>-189, 135	-23.5<br>(59.2)<br>-623, 72	-18.8<br>(40.5)<br>-151, 67	-28.2<br>(73.5)<br>-623, 72
P	---------	**<0.0001**	**0.014**	**<0.0001**	--------	**0.0046**	**0.16**	**0.0003**	---------	**0.0012**	**0.0060**	**0.0024**
HDL=high density lipoprotein cholesterol; LDL=low density lipoprotein cholesterol; LVCF=last value carried forward; trig=triglycerides.												
