# Supplementary figures and images for: Statin Effects on Aggression: Results from the UCSD Statin Study, a Randomized Control Trial
Source: PLoS One. 2015 Jul 1;10(7):e0124451. doi: 10.1371/journal.pone.0124451 (PMC4488854; doi:10.1371/journal.pone.0124451)

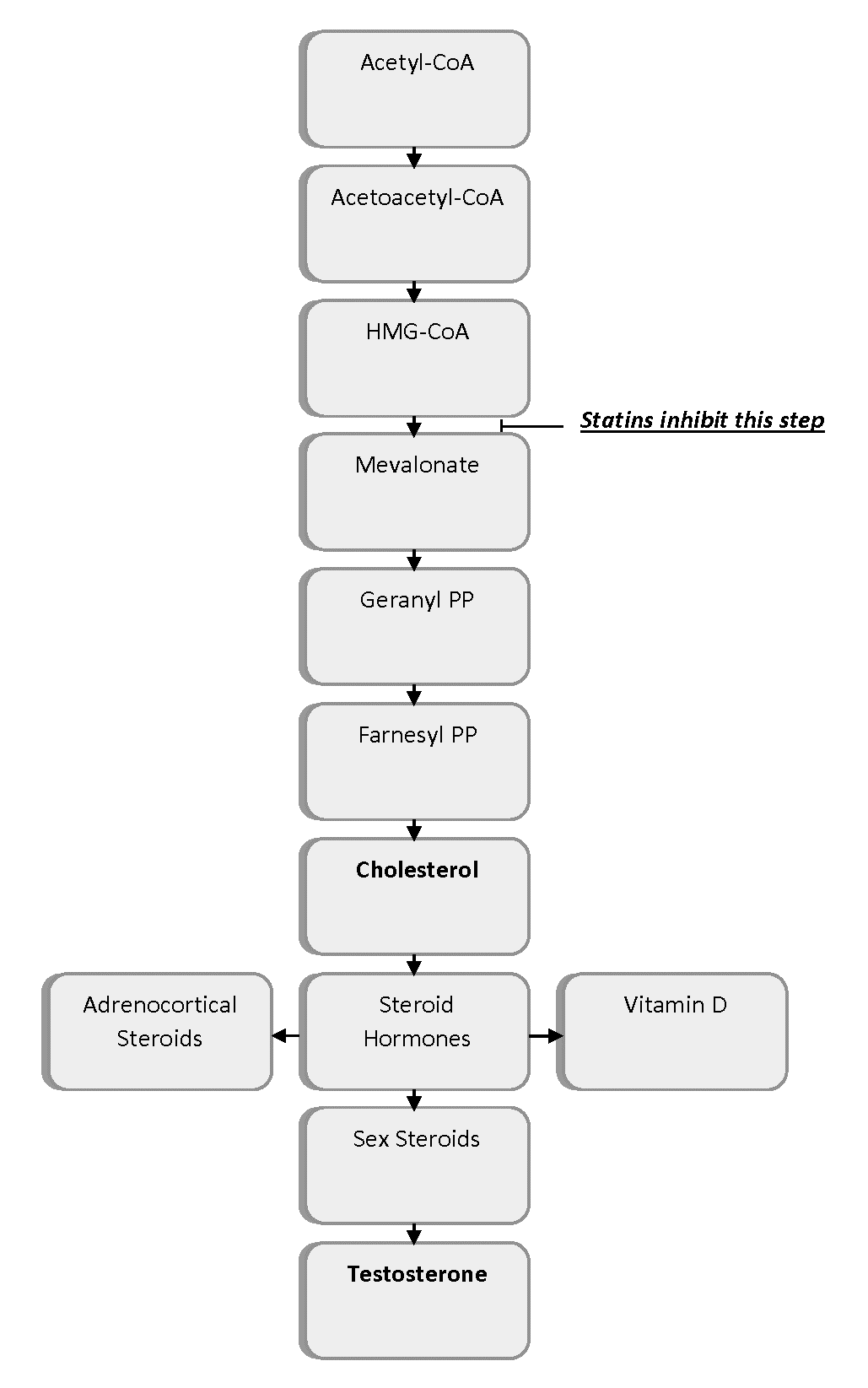

Supplement: S1 Fig — Depicts site of inhibition by statins; shows cholesterol and testosterone as products of the affected pathway. (TIF) [file pone.0124451.s003.tif]

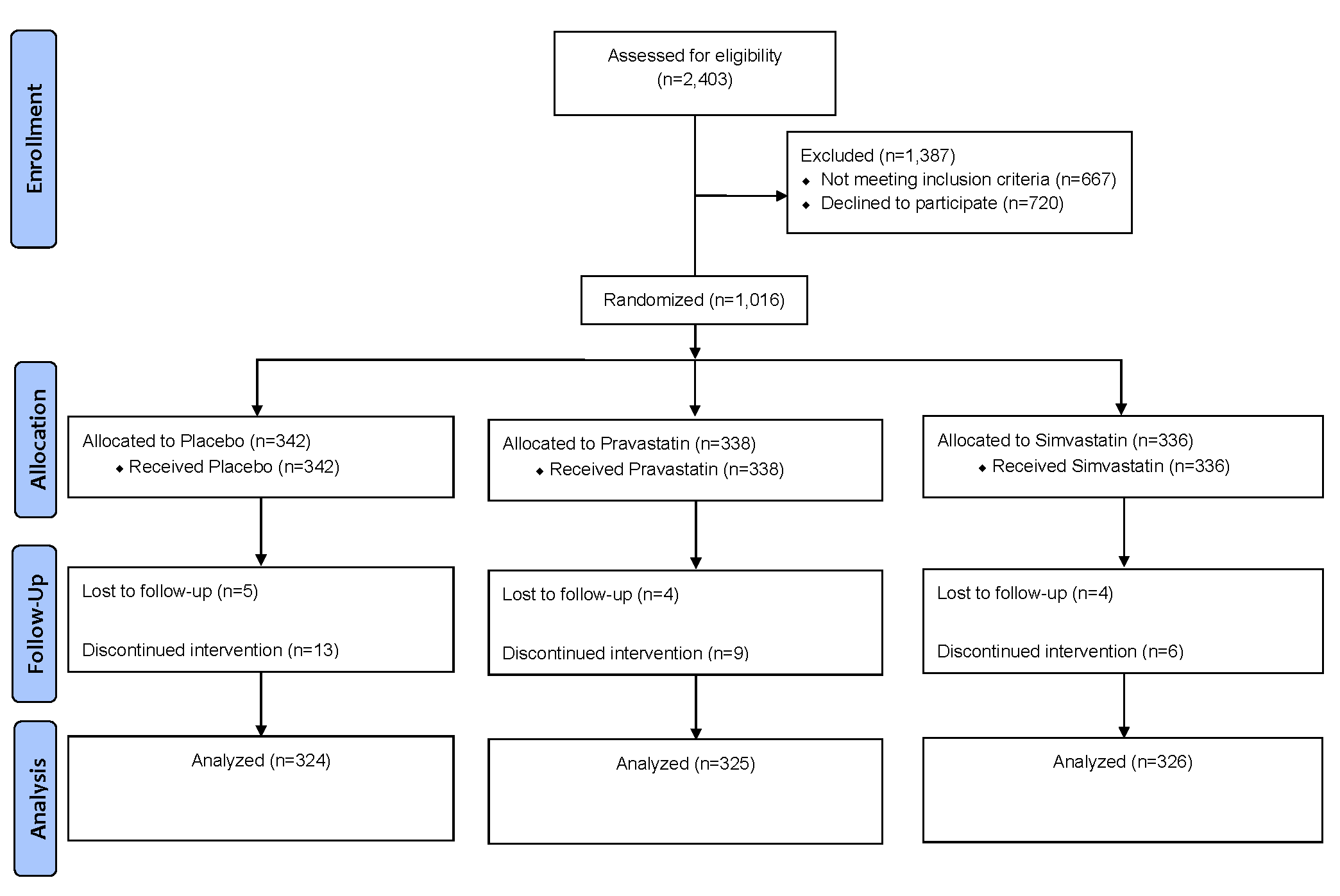

Supplement: S2 Fig — Reasons for participant drops are outlined in S1 Table. (TIF) [file pone.0124451.s004.tif]

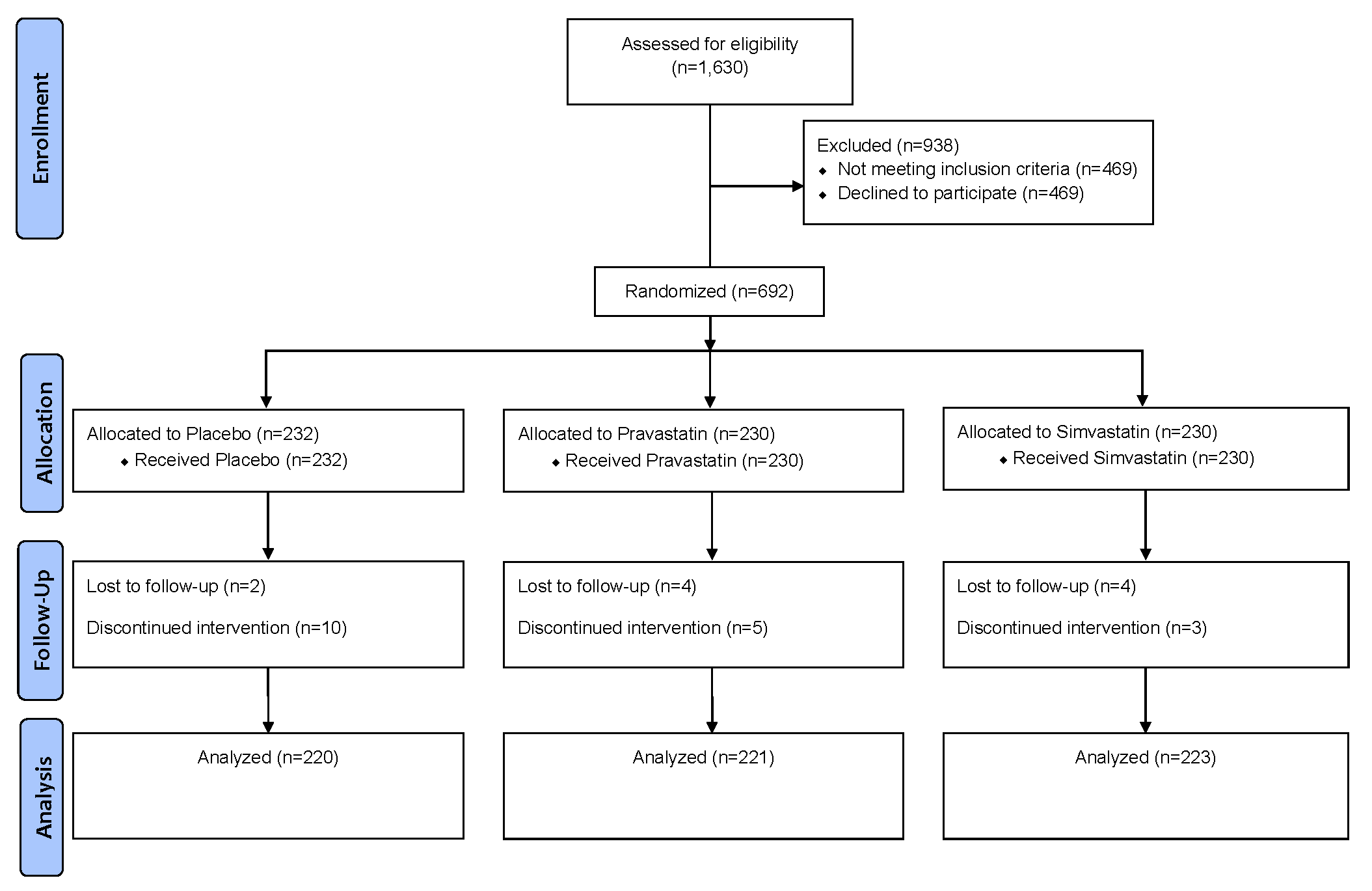

Supplement: S3 Fig — Reasons for participant drops are outlined in S1 Table. (TIF) [file pone.0124451.s005.tif]

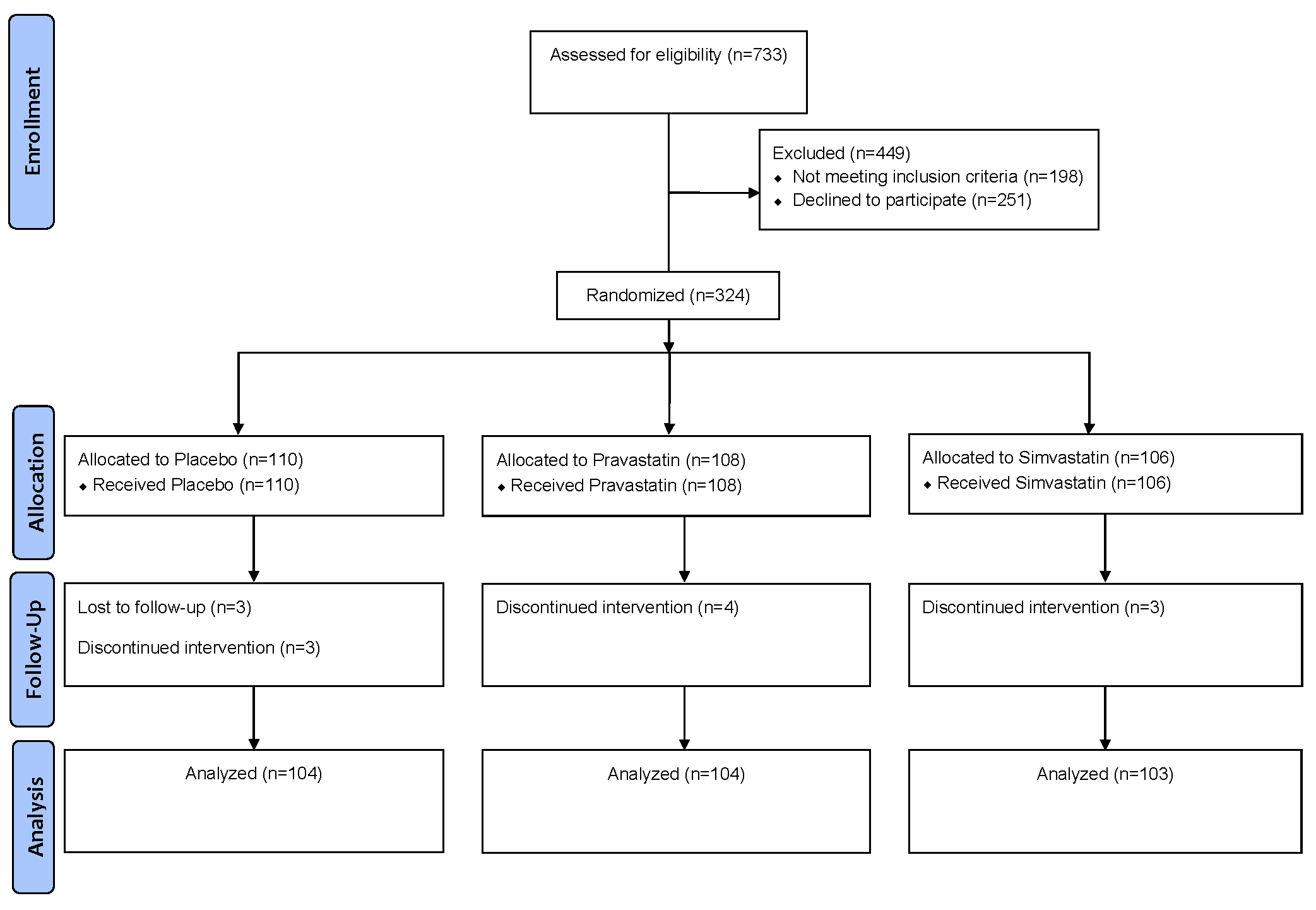

Supplement: S4 Fig — Reasons for participant drops are outlined in S1 Table. (TIF) [file pone.0124451.s006.tif]
