# Supplementary material for: Statin Effects on Aggression: Results from the UCSD Statin Study, a Randomized Control Trial
Source: PLoS One. 2015 Jul 1;10(7):e0124451. doi: 10.1371/journal.pone.0124451 (PMC4488854; doi:10.1371/journal.pone.0124451)
Supplement: S1 Table — (DOC) [file pone.0124451.s007.doc]

S1 Table. Reasons for Study Drops.

|  | **Placebo** | **Pravastatin** | **Simvastatin** |
| --- | --- | --- | --- |
| ***Scheduling*** | 4 | 1 | 0 |
| ***Disliked aspect of study visit*** (e.g. too far, disliked blood draw, questionnaires, etc) | 2 | 1 | 0 |
| ***Physician/PCP recommended they not participate*** | 2 | 0 | 2 |
| ***Surgery*** (or issues related to medical condition predating participation) | 3 | 1 | 2 |
| ***Symptoms*** | 2  a) Felt dizzy  b) Felt uneasiness (which dissipated) and some muscular pain | 4  a) Irritability, depression, suicidal thoughts and nausea after 10 pills  b) Leg pain that subsided following discontinuation  c) Muscle pain attributed to study medication  d) Upper respiratory infection | 1  Muscle pain (also concerned about alcohol plus study medication) |
| ***Miscellaneous*** | 0 | 2  a) Went out of town, forgot to take meds, did not return for follow-up  b) Due to preexisting tracheostomy, could not take study medications without opening capsules | 1  Study psychiatrist felt participation inappropriate (history of depression and suicide attempt) |
