# Supplementary material for: Statin Effects on Aggression: Results from the UCSD Statin Study, a Randomized Control Trial
Source: PLoS One. 2015 Jul 1;10(7):e0124451. doi: 10.1371/journal.pone.0124451 (PMC4488854; doi:10.1371/journal.pone.0124451)
Supplement: S2 Table — (DOC) [file pone.0124451.s008.doc]

S2 Table. Statin Effects on Lipids.

|  | **All** | | | | **Men** | | | | **Women** | | | |
| --- | --- | --- | --- | --- | --- | --- | --- | --- | --- | --- | --- | --- |
|  | Mean (SD)  95%CI | | | | Mean (SD)  95%CI | | | | Mean (SD)  95%CI | | | |
| Completers | N=279 | N=558 | N=280 | N=278 | N=193 | N=368 | N=184 | N=184 | N=86 | N=190 | N=96 | N=94 |
|  | **Placebo** | **Statin** | **Prava** | **Simva** | **Placebo** | **Statin** | **Prava** | **Simva** | **Placebo** | **Statin** | **Prava** | Simva |
| ΔTotal  cholesterol, completer | -2.10 (25.8)  -92, 86 | -51.9  (29.1)  -134, 69 | -46.6 (28.5)  -102, 42 | -57.2 (28.7)  -134, 69 | -3.01 (24.7)  -92, 57 | -51.1 (27.7)  -124, 42 | -46.8 (28.9)  -102, 42 | -55.3 (25.9)  -124, 18 | -0.058 (28.0)  -76, 86 | -53.6 (31.5)  -134, 69 | -46.3 (27.9)  -101, 30 | -61.0 (33.4)  -134, 69 |
| P | **---------** | **<0.0001** | **<0.0001** | **<0.0001** | **---------** | **<0.0001** | **<0.0001** | **<0.0001** | **---------** | **<0.0001** | **<0.0001** | **<0.0001** |
| ΔLDL,  completer | -1.10 (24.0)  -84, 67 | -44.7 (25.1)  -103, 78 | -40.4 (24.4)  -103, 28 | -49.0 (25.1)  -102, 78 | -1.96 (23.4)  -84, 67 | -44.4 (23.8)  -102, 34 | -41.0 (24.2)  -95, 28 | -47.7 (22.9)  -102, 34 | 0.83 (25.4)  -71, 60 | -45.4 (27.6)  -103, 78 | -39.4 (24.9)  -103, 27 | -51.6 (29.0)  -95, 78 |
| P | **---------** | **<0.0001** | **<0.0001** | **<0.0001** | **---------** | **<0.0001** | **<0.0001** | **<0.0001** | **---------** | **<0.0001** | **<0.0001** | **<0.0001** |
| ΔHDL, completer | -1.80 (8.71)  -34, 33 | -1.89  (9.69)  -37, 47 | -1.85 (9.98)  -36, 47 | -1.94 (9.40)  -37, 32 | -1.08 (8.51)  -34, 33 | -1.23 (8.65)  -37, 45 | -1.74 (8.72)  -36, 45 | -0.72 (8.58)  -37, 32 | -3.41 (9.01)  -26, 19 | -3.16 (11.3)  -36, 47 | -2.04 (12.1)  -36, 47 | -4.31 (10.5)  -32, 18 |
| P | **---------** | **0.89** | **0.95** | **0.86** | **---------** | **0.84** | **0.45** | **0.69** | **---------** | **0.86** | **0.39** | **0.54** |
| ΔTrig, completer | -2.09 (58.9)  -333, 275 | -19.5  (60.5)  -623,285 | -13.1 (58.3)  -266,285 | -25.9 (62.0)  -623, 181 | -2.11 (64.1)  -333, 275 | -17.6 (61.0)  -266, 285 | -9.78 (65.4)  -266,285 | -25.3 (55.2)  -192, 181 | -2.03 (45.1)  -118, 118 | -23.2 (59.5)  -623, 72 | -19.3 (41.0)  -151, 67 | -27.1 (73.8)  -623, 72 |
| P | **---------** | **0.0001** | **0.027** | **<0.0001** | **---------** | **0.0053** | **0.25** | **0.0002** | **---------** | **0.0036** | **0.0073** | **0.0072** |
| **LVCF** | **N=322** | **N=645** | **N=324** | **N=321** | **N=219** | **N=440** | **N=220** | **N=220** | **N=103** | **N=205** | **N=104** | **N=101** |
| ΔTotal  cholesterol, LVCF | -1.69 (25.2)  -92, 86 | -51.2 (28.6)  -134, 69 | -46.2 (28.0)  -102, 42 | -56.3 (28.4)  -134, 69 | -2.43 (24.2)  -92, 57 | -50.4 (27.4)  -124, 42 | -46.4 (28.3)  -102, 42 | -54.4 (25.9)  -124, 28 | -0.11 (27.4)  -76, 86 | -53.1 (31.1)  -134, 69 | -45.8 (27.5)  -101, 30 | -60.6 (32.9)  -134, 69 |
| P | **---------** | **<0.0001** | **<0.0001** | **<0.0001** | **---------** | **<0.0001** | **<0.0001** | **<0.0001** | **---------** | **<0.0001** | **<0.0001** | **<0.0001** |
| ΔLDL,  LVCF | -0.98 (24.0)  -84, 67 | -44.5 (24.9)  -105, 78 | -40.2 (24.6)  -105, 28 | -48.9 (24.6)  -102, 78 | -1.24 (22.8)  -84, 67 | -44.1 (23.8)  -105, 34 | -40.5 (24.6)  -105, 28 | -47.6 (22.5)  -102, 34 | -0.44 (26.4)  -71, 65 | -45.5 (27.2)  -103, 78 | -39.4 (24.6)  -103, 27 | -51.8 (28.5)  -95, 78 |
| P | **---------** | **<0.0001** | **<0.0001** | **<0.0001** | **---------** | **<0.0001** | **<0.0001** | **<0.0001** | **---------** | **<0.0001** | **<0.0001** | **<0.0001** |
| ΔHDL, LVCF | -1.51 (8.87)  -34, 33 | -1.46 (9.60)  -37, 47 | -1.51 (9.95)  -36, 47 | -1.40 (9.26)  -37, 32 | -1.08 (8.74)  -34, 33 | -0.92 (8.53)  -37, 45 | -1.56 (8.55)  -36, 45 | -0.28 (8.48)  -37, 32 | -2.42 (9.11)  -26, 21 | -2.60 (11.5)  -36, 47 | -1.40 (12.5)  -36, 47 | -3.84 (10.4) -32, 18 |
| P | **---------** | **0.94** | **0.99** | **0.88** | **---------** | **0.83** | **0.56** | **0.33** | **---------** | **0.89** | **0.50** | **0.30** |
| ΔTrig, LVCF | -1.84 (58.6)  -333, 275 | -19.0 (61.3)  -623,285 | -13.4 (60.1)  -266,285 | -24.6 (62.0)  -623, 181 | -2.27 (62.0)  -333, 275 | -16.9 (62.2)  -266, 285 | -10.9 (67.3)  -266,285 | -22.9 (56.0)  -192, 181 | -0.92 (51.1)  -189, 135 | -23.5 (59.2)  -623, 72 | -18.8 (40.5)  -151, 67 | -28.2 (73.5)  -623, 72 |
| P | **---------** | **<0.0001** | **0.014** | **<0.0001** | **--------** | **0.0046** | **0.16** | **0.0003** | **---------** | **0.0012** | **0.0060** | **0.0024** |
| HDL=high density lipoprotein cholesterol; LDL=low density lipoprotein cholesterol; LVCF=last value carried forward; trig=triglycerides. | | | | | | | | | | | | |
